# Circadian syndrome (CircS) and cognitive trajectory deterioration in middle‐aged and older adults: A national cohort study with causal forest analysis

**DOI:** 10.1002/alz.71300

**Published:** 2026-03-18

**Authors:** Jiangping Ma, Nan Chen, Jingxuan Huang, Linghao Xu, Huifang Shang

**Affiliations:** ^1^ Department of Neurology, Laboratory of Neurodegenerative Disorders, Rare Disease Center, West China Hospital Sichuan University Chengdu China; ^2^ National Clinical Research Center for Geriatric, West China Hospital Sichuan University Chengdu China; ^3^ Department of Endocrinology and Metabolism the Affiliated Hospital of Southwest Medical University Luzhou Sichuan China

**Keywords:** causal forest, circadian syndrome, cognitive trajectory, latent class growth model, longitudinal study

## Abstract

**INTRODUCTION:**

Cognitive impairment is a global health challenge. Circadian syndrome (CircS) extends beyond metabolic syndrome to include sleep and mental health, but its long‐term impact on cognitive trajectories remains unclear.

**METHODS:**

We analyzed 6218 participants from the China Health and Retirement Longitudinal Study (2011–2018). CircS was defined as ≥4 of 7 components. Cognitive trajectories were identified using latent class growth modeling. Associations were assessed via multinomial logistic regression, and heterogeneous effects via causal forest analysis.

**RESULTS:**

Three cognitive trajectories were identified: “persistently high” (40.29%), “persistently moderate” (43.78%), and “persistently low” (15.93%). CircS increased odds of “persistently low” global cognitive (adjusted OR = 1.27, 95%CI:1.06–1.52) and episodic memory (OR = 1.28, 95%CI:1.06–1.55) trajectories. Causal forest identified a vulnerable subgroup: younger age, mild anemia (Hb∼95 g/L), and elevated BMI.

**DISCUSSION:**

CircS is a significant, modifiable risk factor for adverse cognitive trajectories. Screening for CircS could enable early identification and targeted interventions, especially for the identified high‐risk subgroup.

## Highlights

- Novel integration of latent class growth modeling with causal forest machine learning.
- Circadian syndrome increases risk of unfavorable cognitive trajectories by 27%–28%.
- Identification of high‐risk subgroup: younger age, mild anemia, elevated BMI.
- First longitudinal evidence linking circadian syndrome to low cognitive trajectories.
- Circadian syndrome as a modifiable target for early screening and prevention.

## Graphical abstract

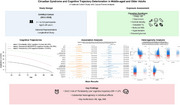

## 1 BACKGROUND

Cognitive impairment presents a growing global public health challenge, particularly for aging populations.^1^, ^2^ As the demographic trend shifts towards an older population structure, the prevalence of cognitive impairment is rising.^3^ Given the current lack of curative treatments, identifying modifiable risk factors for cognitive decline has become a critical strategy for early intervention and prevention.^4^

Metabolic syndrome (MetS) refers to a cluster of concurrent metabolic disorders, mainly including central obesity, hypertension, hyperglycemia, and dyslipidemia.^5^ MetS is a well‐established risk factors for cognitive impairment which is mediated through pathologic changes in the brain, such as reduced brain volume, white matter lesions, and vascular pathology.^6^, ^7^, ^8^ However, the conventional MetS framework does not fully consider other critical risk factors associated with cognitive impairment, such as sleep disturbances and mental health issues. To address this limitation, the concept of Circadian Syndrome (CircS) has been proposed as a more comprehensive disorder characterized by disrupted biological rhythms. CircS encompasses all components of MetS while also integrating additional factors such as reduced sleep duration and depressive symptoms.^9^ There is an increasing consensus that CircS serves as a superior predictor for a range of cardiometabolic diseases compared to MetS alone.^10^, ^11^ Importantly, this enhanced predictive utility extends to cognitive health, with emerging evidence demonstrating a strong correlation between CircS and cognitive impairment.^12^, ^13^ Given the established independent links between sleep, depression and cognitive decline, these findings highlight the potential of CircS as a more integrative and sensitive tool for identifying individuals at high risk for future cognitive impairment.^14^, ^15^

Furthermore, emerging evidence indicates that the association between CircS and cognitive outcomes is not uniform but varies substantially across subgroups. For instance, the detrimental effect of CircS on dementia risk is significantly modified by APOE‐ε4 genotype and smoking status.^16^ In addition, female gender has been associated with a more pronounced cognitive impairment related to CircS.^12^ This heterogeneity suggests that individual susceptibility to the cognitive consequences of circadian and metabolic dysregulation is influenced by a complex interplay of genetic, lifestyle, and sociodemographic factors.

Despite these advancements, the majority of previous research focuses on snapshots of cognition at a certain point in time, rather than its change over time. We still know little about how CircS influences the long‐term trajectory of cognitive function in middle‐aged and elderly individuals. Because cognitive decline is a dynamic process, understanding its trajectory is key for early intervention. The investigation of cognitive trajectories has advanced by utilizing statistics that can find different patterns of changes within a cohort. Many high‐quality longitudinal studies have demonstrated that cognitive trajectories differ between subgroups and possess predictive value for long‐term prognosis, such as conversion to Alzheimer's disease.^17^, ^18^

Causal inference methodologies utilizing real‐world data provide valuable evidence complementary to randomized controlled trials. In observational research, the investigation of heterogeneous treatment effects (HTEs) is fundamental to personalized risk assessment. Wager and Athey advanced this field by developing the causal forest model, an extension of the random forest algorithm, to infer HTEs or conditional average treatment effects (CATEs) from observational data. This innovative approach estimates treatment effects tailored to individual characteristics, enabling personalized causal inference.^19^

Traditional methods for assessing effect heterogeneity, such as interaction terms or subgroup analysis, are often limited by multicollinearity, model dependence, or spurious findings.^20^, ^21^, ^22^, ^23^ In contrast, the causal forest method offers several advantages: it handles high‐dimensional covariates and complex interactions without pre‐specified parametric forms, automatically partitions data based on effect heterogeneity, provides valid confidence intervals (CI), and incorporates cross‐fitting (“honesty”) and out‐of‐bag estimation to mitigate overfitting.^24^, ^25^, ^26^

In this research, we undertook a nationally representative cohort study utilizing four waves of data from the China Health and Retirement Longitudinal Study (CHARLS) spanning 2011–2018, with the objective of investigating the influence of CircS on cognitive aging. Through the application of trajectory‐based modeling and personalized effect estimation, this study furnishes novel insights into the role of CircS as a modifiable risk factor for cognitive decline and proposes targeted prevention strategies for populations at heightened risk.

## 2 METHODS

### 2.1 Study design and population

CHARLS constitutes an ongoing, nationally representative longitudinal survey focusing on adults aged 45 years and older. The primary objective of the study is to gather comprehensive data on sociodemographic characteristics, health status, medical conditions, family structure, and health‐related behaviors. Initiated in 2011, the CHARLS survey has been conducted with follow‐up assessments occurring biennially or triennially. To date, CHARLS has produced four waves of survey data collected in the years 2011, 2013, 2015, and 2018. The research project received approval from the Biomedical Ethics Committee of Peking University (IRB00001052‐11015). Informed consent was obtained from all participants, who also consented to the sharing of their data.

We employed data from the four waves of the CHARLS to identify eligible participants according to specific inclusion and exclusion criteria. Initially, individuals lacking diagnostic information for CircS at the baseline year of 2011 were excluded. Subsequently, participants with cognitive impairment or Parkinson's disease/dementia at baseline were also removed from the study. Additionally, individuals who participated in fewer than two waves of cognitive assessment were excluded. Ultimately, the study comprised 6218 subjects, consisting of 3054 males and 3164 females. A comprehensive flowchart detailing the study design and the inclusion and exclusion of participants is provided in Figure S1.

**RESEARCH IN CONTEXT**

1. **Systematic review**: We reviewed the literature using PubMed and other traditional sources. Previous research has established links between Metabolic Syndrome and cognitive impairment. The novel concept of Circadian Syndrome (CircS) extends this framework to include sleep and mental health, with emerging evidence suggesting its superior predictive value for cardiometabolic diseases and cross‐sectional cognitive status. However, longitudinal evidence on how CircS influences long‐term cognitive trajectories was lacking.
2. **Interpretation**: Our findings demonstrate that CircS is a significant risk factor for adverse long‐term cognitive trajectories, increasing the odds of a “persistently low” trajectory by 27%–28%. Crucially, by applying causal forest analysis, we move beyond average population risks to identify a vulnerable subgroup for whom this effect is most pronounced: younger individuals with mild anemia and elevated BMI. This establishes CircS as a comprehensive, modifiable risk factor and underscores the necessity for personalized cognitive risk assessment.
3. **Future directions**: Future research should: (a) elucidate the precise biological mechanisms, particularly the nonlinear role of hemoglobin, linking CircS to neurodegeneration; (b) develop and test targeted interventions (e.g., circadian rhythm stabilization) through randomized controlled trials in the identified high‐risk subgroup; and (c) validate these findings and the generalizability of the high‐risk profile in diverse ethnic and younger populations.

### 2.2 Assessment of CircS

Venous blood samples were promptly received by the Chinese Center for Disease Control and Prevention in Beijing within two weeks after leaving the CDC station. These samples were immediately stored and frozen at −20°C prior to delivery. After necessary analyses in the laboratory of China Medical University, they were transferred to a deep freezer and maintained at −80°C. In the clinical laboratory of Youanmen, Capital Medical University, the concentrations of biomarkers such as high‐density lipoprotein cholesterol (HDL‐C), low‐density lipoprotein cholesterol(LDL‐C), triglycerides (TG), and glycated hemoglobin (HbA1c) were measured using the enzyme colorimetric method.^12^

Blood pressure was measured three times by trained nurses using a sphygmomanometer, and the average value was calculated. Hypertension was defined as systolic blood pressure ≥140 mmHg and/or diastolic blood pressure ≥90 mmHg or the use of antihypertensive medications.

The Center for Epidemiologic Studies Depression Scale (CES‐D) (10‐item version) was employed to assess depressive symptoms. Depression was defined as a CES‐D score ≥ 10 points.^13^

Anthropometric measurements were conducted by trained staff. Body weight was measured without shoes to an accuracy of 0.1 kg. Height was measured using a vertical stadiometer to an accuracy of 0.1 cm. Waist circumference was measured at the umbilical level to an accuracy of 0.1 cm. Central obesity was defined as a waist circumference ≥85 cm in men and ≥80 cm in women.

Sleep duration was evaluated through personal self‐report. A sleep duration of less than 6 h was classified as reduced sleep duration. It is important to note that sleep health is a multidimensional construct encompassing not only duration but also regularity, timing, efficiency, and quality.^27^ The single‐item measure of sleep duration used here, while practical for large cohort studies and aligned with prior CircS research, may not fully distinguish between efficient short sleep and sleep disruption stemming from circadian misalignment.

The concept of CircS was initially derived from MetS and was assessed based on the following seven components: reduced sleep duration, depression, central obesity, elevated TG (≥150 mg/dL or drug treatment for TG elevation), reduced high‐density lipoprotein (HDL < 40 mg/dL in men; HDL < 50 mg/dL in women; or drug treatment for HDL‐C reduction), hypertension, and elevated fasting blood glucose (≥100 mg/dL or drug treatment for hyperglycemia). Participants with four or more components were diagnosed with CircS.

### 2.3 Assessment of cognitive function

Based on prior research utilizing CHARLS, cognitive function was assessed using the Chinese adaptation of the Mini‐Mental State Examination (MMSE), which evaluates two key dimensions: episodic memory and mental intactness. Participants were presented with a list of 10 unrelated Chinese words and were required to recall them immediately (immediate word recall) and again after a four‐minute interval (delayed word recall). The episodic memory score was determined by averaging the number of words recalled in both the immediate and delayed tasks, with possible scores ranging from 0 to 10. The assessment of mental status included tasks such as serial subtraction of 7 from 100 (up to five iterations), identifying the current date (month, day, and year), naming the day of the week, recognizing the current season, and completing an intersecting pentagon copying test. Responses to these tasks were aggregated to form a mental intactness score, which could range from 0 to 11. The overall cognitive score was calculated by summing the scores for episodic memory and mental intactness, yielding a total score range of 0–21.

### 2.4 Outcome‐cognitive trajectories

The primary outcome was the trajectory of global cognitive scores, and the second outcomes were the trajectory of episodic memory and mental intactness scores. Latent class growth model (LCGM) was used to determine the heterogeneity of cognitive trajectories. LCGM was utilized to identify homogeneous clusters of individuals who follow similar developmental trajectories across time.^28^

### 2.5 Covariates

Interviewers systematically gathered and documented data on sociodemographic characteristics, health‐related behaviors, and medical information utilizing structured questionnaires. The sociodemographic variables encompassed age, gender (categorized as male or female), educational attainment (classified as primary school or below, junior high school to high school, and undergraduate or above), marital status (dichotomized into married and unmarried), and place of residence (rural or urban). Within the marital status classification, individuals identified as “married with spouse present” and “married but not living with spouse” were grouped under “married,” whereas those identified as “separated,” “divorced,” “widowed,” or “never married” were categorized as “unmarried.” Health‐related behaviors assessed included smoking status (yes or no) and alcohol consumption status (yes or no). The medical information collected comprised data on functional restrictions, self‐reported visual and hearing impairments, history of stroke, and heart disease, which included conditions such as heart attack, coronary heart disease, angina pectoris, congestive heart failure, or other cardiac issues. Additionally, measurements of hemoglobin levels and body mass index (BMI) were recorded. Functional restriction was operationally defined as experiencing limitations in any of the five activities of daily living (ADL), namely bathing, dressing, eating, transferring in and out of bed, and toileting. BMI was calculated as the weight in kilograms divided by the square of the height in meters and was categorized as follows: <18.5, 18.5–23.9, 24.0–27.9, and ≥28.0 kg/m^2^.

### 2.6 Statistical analysis

Continuous normally distributed variables were described using means and standard deviations. Non‐normally distributed continuous variables were described using medians and interquartile ranges. Categorical variables were described using frequencies and percentages. one‐way analysis of variance, rank sum tests, or chi‐square tests were used to compare baseline differences between groups.

#### 2.6.1 Identification of cognitive trajectories

To capture the heterogeneity in cognitive change over time, we identified distinct cognitive trajectories using the LCGM. This method groups individuals into homogeneous subpopulations (latent classes) that share similar developmental paths of global cognitive scores across the study waves. Age in years was used as the timescale. We tested models with 2–5 trajectory classes, employing both linear and quadratic functions. The optimal number and shape of trajectories were determined based on the following criteria^29^: (1) the lowest sample size‐adjusted Bayesian information criterion (SABIC); (2) an average posterior probability of assignment (AvePP) for each trajectory class greater than 0.7, indicating adequate class separation; and (3) each trajectory class comprising no less than 5% of the total sample to ensure clinical relevance. Detailed statistical methods were provided in the Supplementary File: Supplementary Method 1.

#### 2.6.2 Association analysis between CircS and cognitive trajectories

Multinomial logistic regression model was used to estimate the association of Circs with the trajectories of the cognitive function measures. Odds ratios (OR) and the corresponding 95% CI were reported. To preliminarily assess potential effect modification and to examine the consistency of the CircS‐cognitive trajectory association across key demographic and clinical subgroups, we performed stratified analyses by each of the following baseline covariates: sex (male, female), age group (<60, ≥60 years), residence (urban, rural), educational level (primary school or below, junior high school to high school, undergraduate or above), marital status (married, unmarried), smoking status (yes/no), drinking status (yes/no), BMI category (<18.5, 18.5–23.9, 24.0–27.9, ≥28.0 kg/m^2^), history of stroke (yes/no), history of heart disease (yes/no), ADL restriction (yes/no), visual impairment (yes/no), hearing impairment (yes/no), and hemoglobin level (split at the median into high/low groups). These stratified analyses served a dual purpose: (1) as an initial, descriptive exploration of heterogeneity in association strength across subgroups, and (2) as robustness checks to ensure the primary association was not driven by a specific subpopulation. Subsequently, to formally test for statistically significant effect modification, we introduced multiplicative interaction terms (e.g., CircS × sex, CircS × age group) individually into the fully adjusted multinomial logistic regression model. The significance of the interaction term was assessed using the likelihood ratio test.

#### 2.6.3 Causal forest analysis

To further investigate the HTEs of CircS on cognitive trajectories, we employed a causal forest machine learning approach. We constructed three separate causal forest models for each cognitive trajectory outcome (persistently low, moderate, and high). The analysis included 14 pre‐specified covariates: age, gender, residence, education, marital status, smoking, drinking, heart disease, stroke, hemoglobin, BMI, visual impairment, hearing impairment, and ADL ability. Each forest comprised 10,000 trees and utilized honest estimation (50% of the data for splitting, 50% for estimation) to minimize overfitting.

We assessed model calibration and estimated the average treatment effect (ATE) alongside Individual Treatment Effects (ITEs) to quantify effect heterogeneity. Variable importance was analyzed to identify key moderators of the CircS effect on cognitive trajectories. All analyses were performed using R version 4.4.2 with the grf package, and a random seed was set for reproducibility. Sensitivity analyses with fewer trees were used to confirm the robustness of our results. Detailed statistical methods were provided in the Supplementary File: Supplementary Method 2.

#### 2.6.4 Sensitivity analysis

1. Inverse Probability Weighting (IPW): We conducted IPW analysis to address potential confounding and validate our primary findings. Propensity scores were estimated using logistic regression with CircS as the outcome and all baseline covariates as predictors. Stabilized weights were applied to create a balanced pseudo‐population. Weighted multinomial logistic regression was used to estimate the association between CircS and cognitive trajectories.
2. Linear Mixed‐Effects Models (LMMs): We fitted LMMs to directly model the repeated measures of global cognitive scores as a continuous outcome over time. This approach treats cognitive decline as a continuous process and does not presuppose the existence of discrete trajectory classes. The core model included fixed effects for CircS status (present/absent), time (in years from baseline), and their interaction term (CircS × Time). A significant interaction term would indicate that the rate of cognitive decline (slope) differs between individuals with and without CircS. The models were adjusted for the same set of baseline covariates used in the multinomial logistic regression.
3. Continuous CircS Score Analysis: As an additional sensitivity analysis, we examined the association between a continuous CircS score (range: 0–7) and cognitive trajectories using multinomial logistic regression. This approach treats CircS as a dimensional construct rather than a categorical diagnosis, allowing us to investigate potential dose–response relationships. Both crude and fully adjusted models were fitted using the same covariate sets as the primary binary analysis.
4. Sensitivity Analysis with Additional Covariates: To further address concerns regarding potential residual confounding, an extended sensitivity analysis was performed. The original set of covariates was supplemented with five additional baseline variables for model refitting: physical activity level (categorized as low, moderate, or high), frequency of social activities (categorized as none, moderate, or high), use of sleep medication (yes/no), use of antidepressant medication (yes/no), and habitual napping. Both the multinomial logistic regression models and the causal forest analysis were repeated using this extended covariate set.
5. Sensitivity Analysis Using Multiple Imputation: To assess potential selection bias arising from missing CircS data at baseline, we conducted a sensitivity analysis using multiple imputation. All missing components defining CircS, along with other baseline covariates, were imputed. The complete analytical workflow—including cognitive trajectory identification, multinomial logistic regression, and causal forest analysis—was then performed on the imputed data. The pooled results were compared to the primary complete‐case analysis to evaluate the robustness of the findings.
6. Sensitivity Analysis Examining Collinearity and a Modified CircS Definition: To further assess the robustness of BMI as a potential effect moderator, two supplementary analyses were conducted. First, variance inflation factors (VIFs) were calculated for all covariates in the causal forest model to examine potential multicollinearity. Second, we repeated the primary causal forest analysis using a modified CircS definition, which excluded the central obesity component. In this analysis, CircS was defined as the presence of ≥3 out of the remaining 6 components. All other model specifications were kept identical to the primary analysis.

#### 2.6.5 Extended analysis

We conducted an extended analysis using person‐specific rates of cognitive change as the outcome. For each participant, a linear regression model was fitted with the global cognitive score as the dependent variable and time since baseline (in years) as the independent variable. The individual‐specific slope from this model represented the rate of cognitive change. Participants were then classified into a binary transition outcome: “Cognitive Decline” (slope < 0) versus “Cognitive Stability or Improvement” (slope ≥ 0). The association between CircS and this binary transition outcome was examined using a logistic regression model, adjusted for the same baseline covariates as in the primary analysis. A causal forest analysis was subsequently performed to estimate the heterogeneous effects of CircS on the risk of “Cognitive Decline.”

The above statistical analyses were conducted using SPSS 25.0 and R 4.4.2. A two‐sided *p*‐value < 0.05 indicated a significant result.

## 3 RESULTS

### 3.1 Population characteristics

In the study, from the initial cohort of 17,708 participants in CHARLS at baseline, individuals lacking CircS information (*n* = 7313) were excluded. Subsequently, participants with cognitive impairment (*n* = 678, Figure S2 presents the age‐specific corresponding cognitive impairment thresholds) or diagnosed with Parkinson's disease/dementia (*n* = 91) were also excluded. Additionally, those with fewer than two waves of cognitive assessment data (*n* = 3408) were removed from the analysis. Ultimately, the final analytical sample comprised 6218 subjects. Figure S1 illustrates the study design and provides a detailed flowchart of inclusion and exclusion criteria of participants.

Table 1 summarizes the baseline characteristics of the included participants. The mean age of the sample was 57.35 years (SD = 8.65), with 3054 (49.1%) being male. Statistically significant differences were observed in the age, sex, place of residence, educational level, marital status, smoking and drinking history, ADL ability, BMI, history of stroke and heart disease, Visual impairment, and hemoglobin levels between participants with and without CircS.

**TABLE 1:** Baseline characteristics (described based on the presence or absence of circadian syndrome).

Variable	All (*N* = 6218)	No CircS (*N* = 4079)	CircS (*N* = 2139)	*p*.overall
Age (years), mean ± SD	57.35 ± 8.65	57.05 ± 8.67	57.91 ± 8.59	0.0002
Sex, *n* (%)				<0.0001
Male sex	3054 (49.1%)	2254 (55.3%)	800 (37.4%)	
Female sex	3164 (50.9%)	1825 (44.7%)	1339 (62.6%)	
Residence, *n* (%)				
Urban	2186 (35.2%)	1362 (33.4%)	824 (38.5%)	0.0001
Rural	4032 (64.8%)	2717 (66.6%)	1315 (61.5%)	
Educational level, *n* (%)				
Below primary school	4137 (66.5%)	2641 (64.7%)	1496 (69.9%)	0.0002
Secondary to vocational school	2024 (32.6%)	1400 (34.3%)	624 (29.2%)	
University and above	57 (0.9%)	38 (0.9%)	19 (0.9%)	
Marital status, *n* (%)				
Unmarried	483 (7.8%)	262 (6.4%)	221 (10.3%)	<0.0001
Married	5735 (92.2%)	3817 (93.6%)	1918 (89.7%)	
Smoking status, *n* (%)				
No	3715 (59.7%)	2267 (55.6%)	1448 (67.7%)	<0.0001
Yes	2503 (40.3%)	1812 (44.4%)	691 (32.3%)	
Drinking status, *n* (%)				
No	3729 (60.0%)	2311 (56.7%)	1418 (66.3%)	<0.0001
Yes	2489 (40.0%)	1768 (43.3%)	721 (33.7%)	
Restriction on ADL, *n* (%)				
No	5453 (87.7%)	3680 (90.2%)	1773 (82.9%)	<0.0001
Yes	765 (12.3%)	399 (9.8%)	366 (17.1%)	
History of stroke, *n* (%)				
No	6104 (98.2%)	4021 (98.6%)	2083 (97.4%)	0.0012
Yes	114 (1.8%)	58 (1.4%)	56 (2.6%)	
Heart disease, *n* (%)				
Yes	620 (10.0%)	329 (8.1%)	291 (13.6%)	<0.0001
No	5598 (90.0%)	3750 (91.9%)	1848 (86.4%)	
BMI (kg/m^2^), *n* (%)				
<18.5	343 (5.5%)	312 (7.6%)	31 (1.4%)	<0.0001
18.5–23.9	3235 (52.0%)	2503 (61.4%)	732 (34.2%)	
24.0–27.9	1915 (30.8%)	990 (24.3%)	925 (43.2%)	
≥28.0	725 (11.7%)	274 (6.7%)	451 (21.1%)	
Visual impairment, *n* (%)				
No	5876 (94.5%)	3879 (95.1%)	1997 (93.4%)	0.0052
Yes	342 (5.5%)	200 (4.9%)	142 (6.6%)	
Hearing impairment, *n* (%)				
No	5796 (93.2%)	3820 (93.7%)	1976 (92.4%)	0.0658
Yes	422 (6.8%)	259 (6.3%)	163 (7.6%)	
Hb (g/L), mean ± SD	90.58 ± 8.53	90.83 ± 8.63	90.10 ± 8.31	0.0013

SD: standard deviation; BMI: body mass index; Hb: Hemoglobin; CircS: Circadian syndrome.

**FIGURE 1:**
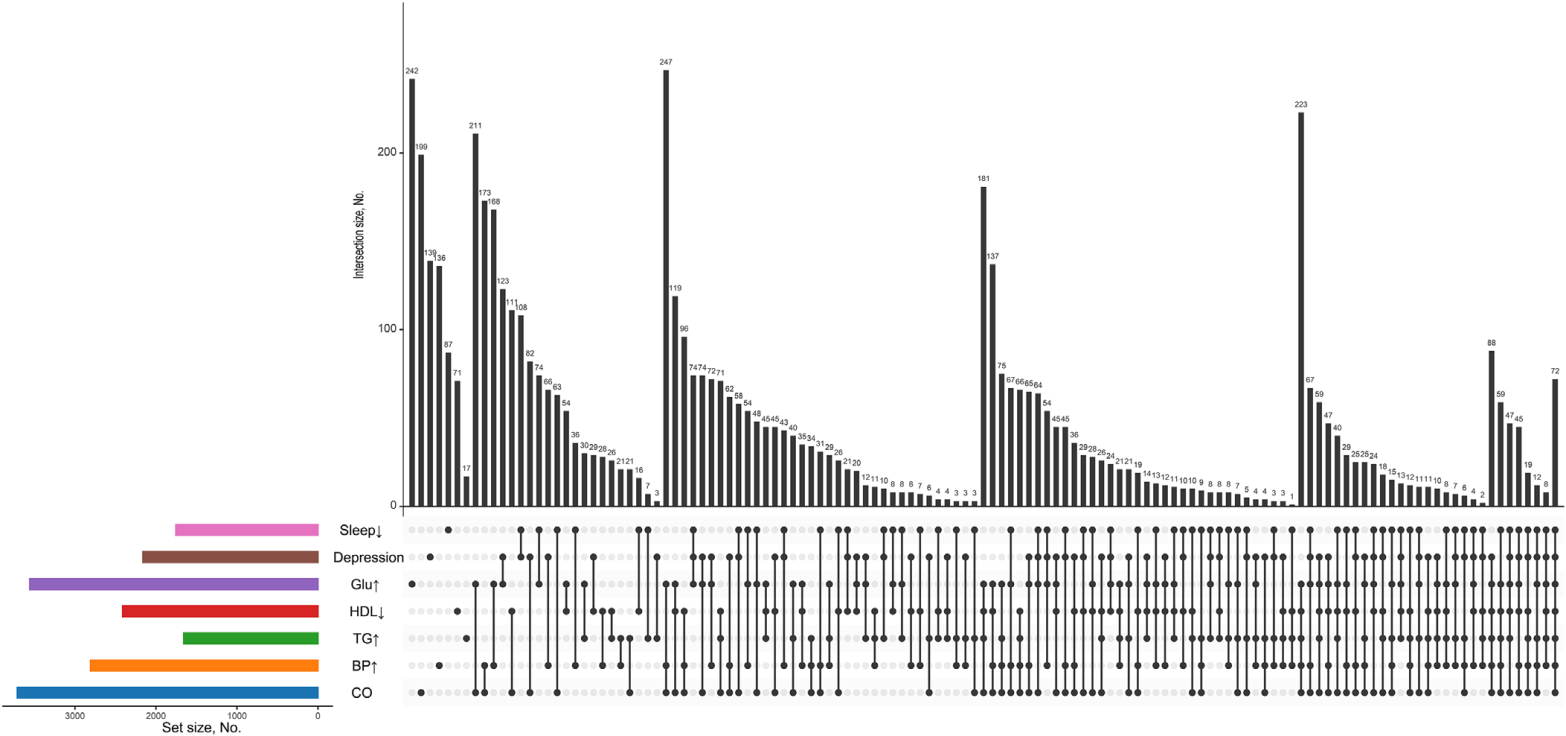
UpSet plot displaying the frequency and combinations of the seven components defining circadian syndrome (Sleep↓, reduced sleep duration; Glu↑, elevated fasting blood glucose; HDL↓, reduced high‐density lipoprotein; TG↑, elevated triglycerides; BP↑, hypertension; CO, central obesity).

The co‐occurrence patterns of the seven CircS components are presented in Figure 1. The most frequent combinations involved central obesity with other metabolic components, while the combination of reduced sleep duration and depressive symptoms co‐occurred in a distinct subgroup.

Figure S3 presents the distribution of global cognitive scores at baseline.

### 3.2 Estimated cognitive aging trajectories

**FIGURE 2:**
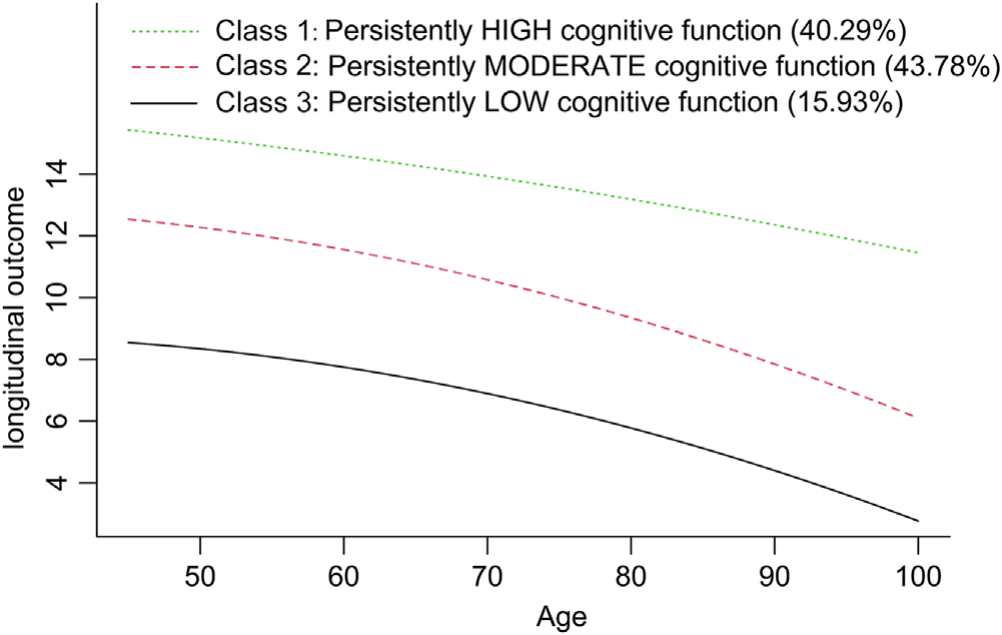
Mean trajectories of global cognitive scores by increasing age among participants.

We evaluated the optimal number of cognitive function trajectories necessary to account for the variability in global cognitive scores within this population (refer to Table S1). The BIC was lowest for the model comprising four trajectories (BIC =  96,886.26); however, the average posterior probability for this model was below 0.7. Consequently, we determined that the GBTM approach with three trajectories was the most suitable. Figure 2 illustrates three longitudinal patterns of cognitive function, categorized by current age at each assessment, based on global cognitive scores: Class 1, “Persistently High” (*n* = 2505, 40.29%); Class 2, “Persistently Moderate” (*n* = 2722, 43.78%); and Class 3, “Persistently Low” (*n* = 991, 15.93%). The trajectories for cognitive function domains are presented in the Figure S4.

### 3.3 Trajectory sub‐population characteristics

As illustrated in Table S2, the baseline characteristics are presented in accordance with global cognitive trajectories. A range of significant statistical differences were identified in the age, gender, place of residence, educational attainment, marital status, smoking history, drinking history, ADL ability, BMI, hearing impairment, and visual impairment among the three cognitive trajectory groups.

### 3.4 Associations between CircS and cognitive trajectories

The distribution of cognitive trajectories, stratified by CircS status, is depicted in Figure S5. A chi‐square test revealed a significant association between CircS status and cognitive trajectory membership (*p* < 0.001). Visually, individuals with CircS were proportionally more likely to belong to the “Persistently Low” trajectory and less likely to belong to the “Persistently High” trajectory compared to those without CircS. This preliminary unadjusted association necessitated further investigation using multivariable‐adjusted models.

Table 2 presents the findings from the multinomial regression analysis examining the association between CircS and cognitive trajectory membership. Compared to participants without CircS, adults with CircS exhibited poorer cognitive trajectories, with multivariable‐adjusted OR and 95% CI for the “Persistently Low” trajectories of global cognitive function being 1.27 [1.06–1.52]. Similarly, adults with CircS demonstrated worse cognitive trajectories, with multivariable‐adjusted OR (95% CI) for the “Persistently Low” trajectories of episodic memory being 1.28 [1.06–1.55].

**TABLE 2:** Multinomial logistic regression analysis for the associations of CircS with the membership to cognitive function trajectory group.

	Persistently low (vs. persistently high)		Persistently moderate (vs. persistently high)	
	OR (95% CI)^*^	p value	OR (95% CI)^*^	p value
Global cognitive scores				
Crude model	1.40[1.20–1.63]	<0.001	1.08[0.96–1.21]	0.189
Adjusted model^*^	1.27[1.06–1.52]	0.009	1.10[0.97–1.26]	0.132
Mental intactness scores				
Crude model	1.31[1.11–1.55]	0.002	1.14[1.02–1.28]	0.026
Adjusted model^*^	1.18[0.97–1.43]	0.099	1.09[0.96–1.25]	0.195
Episodic memory scores				
Crude model	1.21[1.02–1.43]	0.027	1.10[0.95–1.28]	0.209
Adjusted model^*^	1.28[1.06–1.55]	0.012	1.14[0.96–1.35]	0.135

95%CI: 95% confidence interval;

* Adjusted for age at baseline (continuous), gender (male, female), education (primary school or below, junior high school to high school, undergraduate or above), marital status (married, unmarried), residence (urban, rural), smoking (yes, no), drinking (yes, no), body mass index (<18.5, 18.5–23.9, 24.0–27.9, ≥28.0 kg/m^2^), restriction on activities of daily living (yes, no), visual impairment (yes, no), hearing impairment (yes, no), stroke (yes, no), heart disease (yes, no), and hemoglobin (continuous).

**FIGURE 3:**
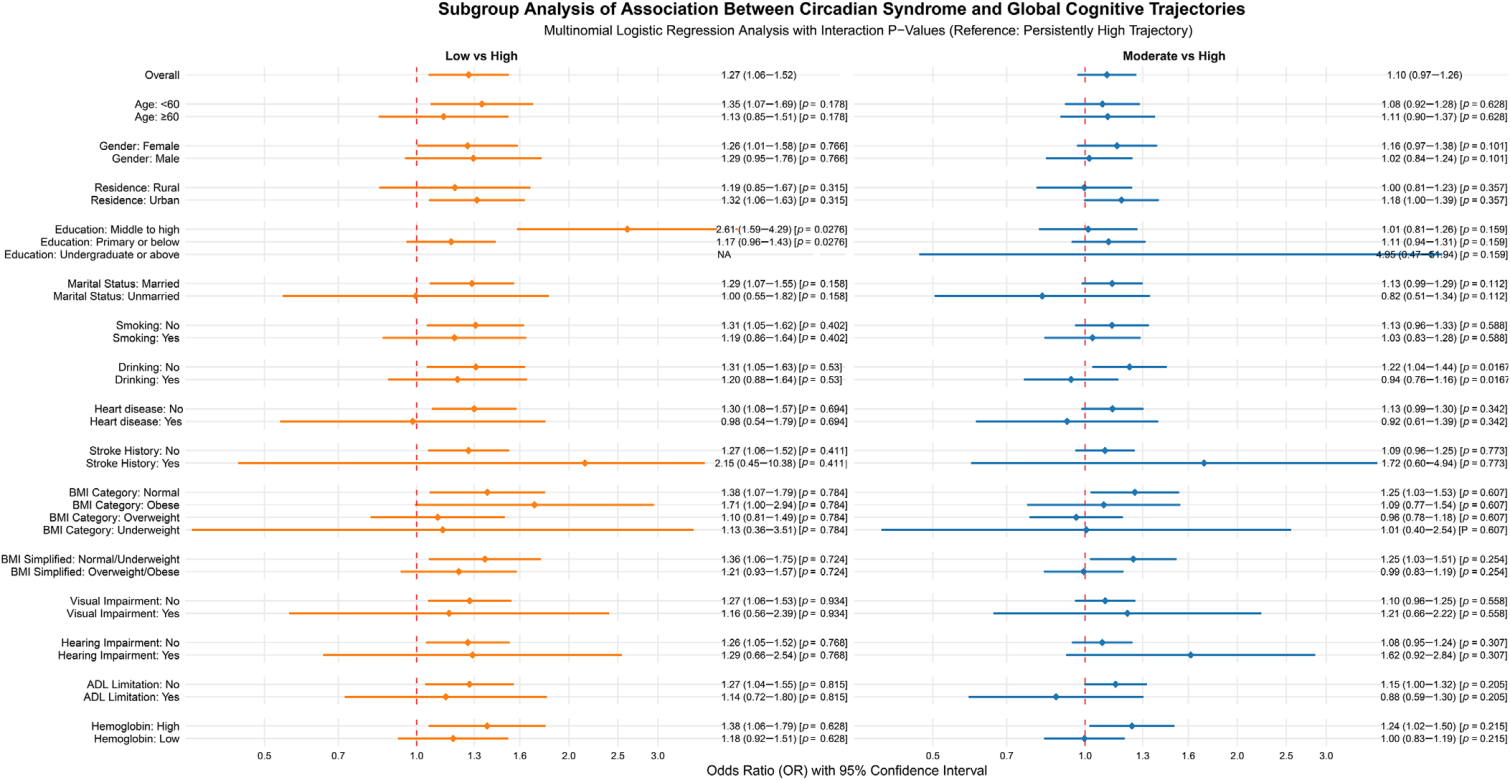
Stratified analysis of the association between circadian syndrome and global cognitive trajectory membership across all covariates, with interaction *p*‐values.

Comprehensive analyses of interactions identified several significant effect modifiers across various cognitive domains and trajectory comparisons. In terms of global cognitive trajectories, significant interactions were detected between CircS and education level (*p* = 0.0276) in the comparison between “persistently low” and “persistently high” trajectories, as well as between CircS and drinking status (*p* = 0.0167) in the comparison between “persistently moderate” and “persistently high” trajectories (see Figure 3). Regarding domain‐specific trajectories, episodic memory (refer to Figure S6) exhibited significant interactions with drinking status (*p* = 0.0337) and heart disease (*p* = 0.04) in the comparison between “persistently low” and “persistently high” trajectories, while mental intactness (refer to Figure S7) showed a significant interaction with heart disease (*p* = 0.0309) in the comparison between “persistently moderate” and “persistently high” trajectories. These findings indicate that the cognitive effects of CircS are influenced by distinct factors depending on the specific cognitive domain and the severity of the trajectory.

### 3.5 Casual forest analysis

We employed causal forest analysis to estimate the HTEs of CircS on distinct cognitive trajectories. The analysis included 6218 participants with 2139 individuals diagnosed with CircS and 4079 controls.

#### 3.5.1 ATEs

**FIGURE 4:**
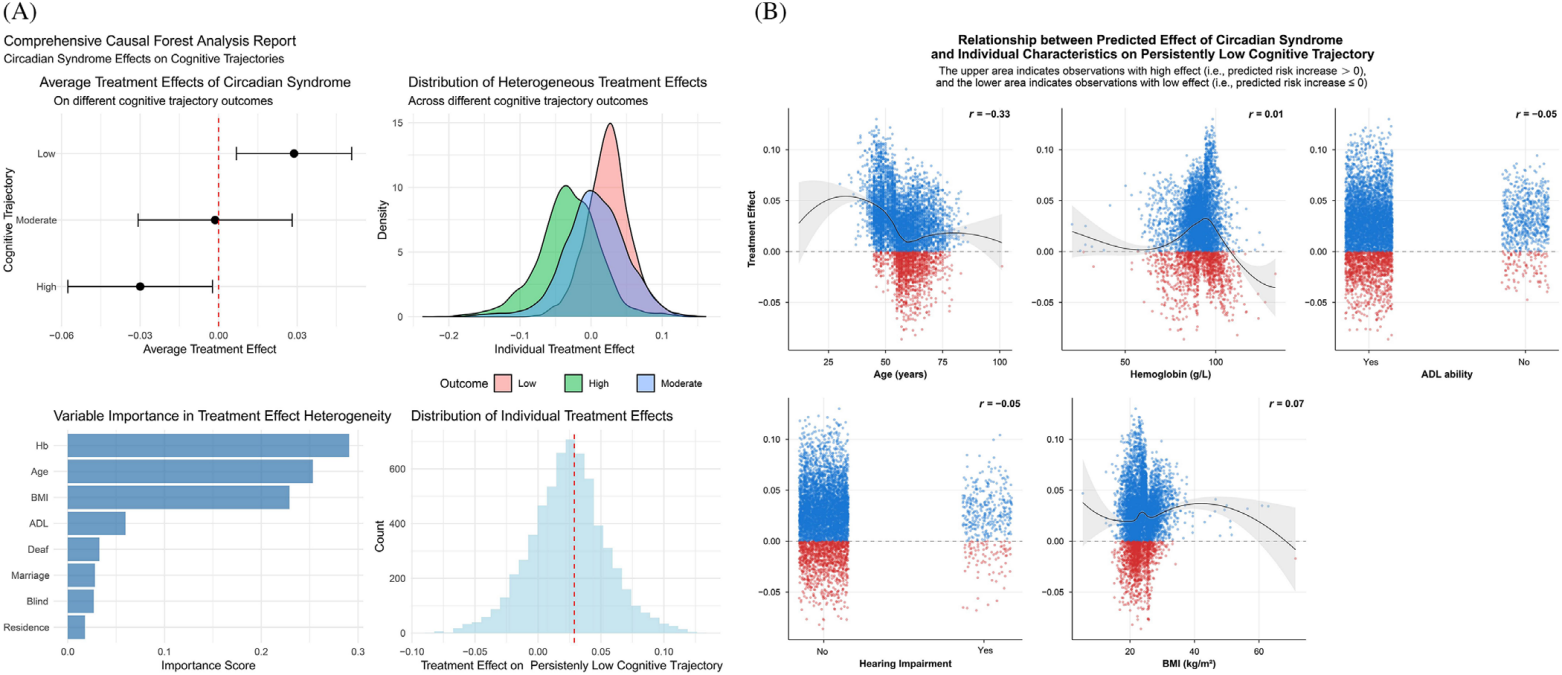
Heterogeneous treatment effects of circadian syndrome on cognitive trajectories: (A) causal forest analysis of circadian syndrome effects on cognitive trajectories; (B) the predictive impact of circadian syndrome on persistently low cognitive trajectories and its relationship with age, hemoglobin level, ADL ability, hearing impairment, and body mass index.

The ATE of CircS varied across different cognitive trajectories (Figure 4A). For the persistently low cognitive trajectory, the ATE was 0.029 (95% CI: 0.007–0.051), indicating a statistically significant association. The persistently moderate cognitive trajectory showed an ATE of −0.002 (95% CI: −0.031 to 0.028), while the persistently high cognitive trajectory demonstrated an ATE of −0.029 (95% CI: −0.057 to −0.002).

#### 3.5.2 Heterogeneity of treatment effects

Substantial heterogeneity in ITEs was observed in persistently low cognitive trajectory (Figure 4A). The standard deviation of ITEs for persistently low cognitive trajectory was 0.0247, ranging from −0.0639 to 0.1063. The standard deviation of ITEs for persistently moderate cognitive trajectory was 0.0429, ranging from −0.1994 to 0.1478 (Figure S8). The standard deviation of ITEs for persistently high cognitive trajectory was 0.0431, ranging from −0.2279 to 0.1454 (Figure S8).

#### 3.5.3 Variable importance in treatment effect heterogeneity

Variable importance analysis identified key moderators of treatment effect heterogeneity for persistently low cognitive trajectory risk (Figure 4A). The most influential variables were: (1) Hemoglobin (Hb)—Importance score: 0.287; (2) Age—Importance score: 0.252; (3) BMI—Importance score: 0.236; (4) ADL—Importance score: 0.058; (5) Hearing impairment (Deaf)—Importance score: 0.030. These variables accounted for the majority of heterogeneity in how CircS affects cognitive decline risk. Variable importance analysis also identified key moderators of treatment effect heterogeneity for persistently moderate and high cognitive trajectories. The top three variables were similarly BMI, Hb, and Age (see Figure S8).

#### 3.5.4 Relationship between predicted treatment effects and individual characteristics

The analysis of HTEs revealed distinct patterns between predicted effect of CircS and key individual characteristics for persistently low cognitive trajectory (Figure 4B). The visualization clearly demarcated high‐effect (predicted risk increase > 0) and low‐effect (predicted risk increase ≤ 0) subgroups across all covariate distributions.

A moderate negative correlation was observed between age and treatment effects (*r* = −0.33), indicating that older individuals derived significantly less treatment effects from CircS. The treatment effects showed a clear decreasing trend with advancing age, with the highest treatment effects observed in younger participants. While the simple linear correlation between Hb and the CircS effect was negligible (*r* = 0.01), the causal forest model, capable of capturing complex, nonlinear dependencies, identified Hb as the most important moderator (variable importance score: 0.287). This apparent discrepancy was resolved by visualizing the relationship, which uncovered a distinct U‐shaped pattern. Specifically, the treatment effects of CircS on persistently low cognitive trajectory were most pronounced for individuals with Hb levels concentrated in a mild‐anemia range (approximately 95 g/L), whereas those at both the lower and upper extremes of the Hb distribution exhibited relative resilience. This nonlinear pattern underscores that Hb remains a variable of paramount importance in moderating the cognitive impact of CircS, even in the absence of a strong linear association, and highlights a specific “metabolic‐vulnerable” phenotypic window at middling Hb levels. A weak negative correlation emerged between ADL, hearing impairment and influence (*r* = −0.05), with a slight tendency for individuals without ADL ability or with hearing impairment to experience greater treatment effects against cognitive decline. A weak positive correlation was observed between BMI and the ITE of CircS (*r* = 0.07), suggesting a tendency for individuals with a higher BMI to experience a slightly greater detrimental effect from CircS on the risk of a persistently low cognitive trajectory.

Supplementary analyses examined the heterogeneity of CircS effects on the “persistently moderate” and “persistently high” cognitive trajectories (Figure S9). Interpretation of the “persistently moderate” trajectory was limited, as it was defined relative to both the high and low trajectory classes. For the “persistently high” trajectory, the moderating effect of age was minimal (*r* = −0.02), indicating that the adverse association between CircS and maintaining high cognitive function was largely consistent across the studied age range. Hb exhibited a near U‐shaped relationship with the CircS effect, with peak susceptibility observed around 95 g/L, consistent with findings from the low‐trajectory analysis. BMI also showed a U‐shaped moderating effect, with the strongest negative effect of CircS on maintaining a high trajectory occurring at approximately 24 kg/m^2^. While this pattern is not fully aligned with the low‐trajectory results, it underscores the importance of maintaining a normal BMI for cognitive health.

#### 3.5.5 Robustness and calibration

Model calibration tests confirmed adequate fit for all three cognitive trajectories. The heterogeneity coefficients were 1.01 (*p* = 0.01) for the persistently low cognitive trajectory, 0.82 (*p* = 0.24) for the persistently moderate cognitive trajectory, and 0.98 (*p* = 0.01) for the persistently high cognitive trajectory. Sensitivity analyses using reduced tree numbers (5000 trees) yielded consistent results, with ATE differences of 0.0001, 0.0007, and 0.0005 for the low, moderate, and high cognitive trajectories, respectively.

### 3.6 Sensitivity analysis

#### 3.6.1 IPW

The IPW analysis demonstrated adequate covariate balance after weighting, with all standardized mean differences below 0.1 (Figure S10). The distribution of inverse probability weights showed appropriate specification with minimal extreme weights (Figure S11).

In the weighted analysis, the association between CircS and cognitive trajectories remained consistent with our primary findings. For the low versus high trajectory comparison, the weighted OR was 1.31 (95% CI: 1.08–1.56). For the moderate versus high trajectory comparison, the weighted OR was 1.09 (95% CI: 0.96–1.25). The distribution of cognitive trajectories before and after weighting demonstrated successful balancing of the study population (Figure S12).

These IPW results confirm the robustness of our primary multinomial logistic regression findings, providing additional evidence for the association between CircS and unfavorable cognitive trajectories.

#### 3.6.2 LMMs

The sensitivity analysis using LMMs revealed distinct patterns of cognitive decline associated with CircS status over time (Figure S13). We observed significant interaction effects between CircS and age for both global cognitive scores (*β* = −0.02, *p* = 0.003) and episodic memory (*β* = −0.01, *p* = 0.02), indicating that the presence of CircS was associated with an accelerated rate of cognitive decline in these domains as age increased.

In contrast, no significant interaction was found between CircS and age for mental intactness scores (*β* = −0.01, *p* = 0.054), suggesting that the decline in this cognitive domain might follow a similar trajectory regardless of CircS status.

Visual inspection of the predicted trajectories clearly demonstrates that participants with CircS (red line) exhibited steeper declines in global cognition and episodic memory compared to those without CircS (blue line). This pattern was consistent with our primary analyses using trajectory modeling.

#### 3.6.3 Continuous CircS score analysis

When analyzing CircS as a continuous variable, we observed differences from the primary binary analysis (Table S3). For the low versus high trajectory comparison, each one‐unit increase in the CircS score was associated with a 16% increase in odds (adjusted OR = 1.16, 95% CI: 1.05–1.18), consistent with the direction of our primary analysis but suggesting a more graded dose–response relationship.

**FIGURE 5:**
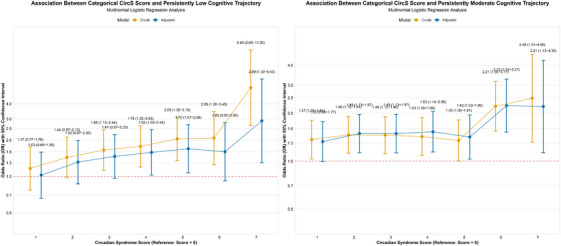
Dose–response relationships between continuous circadian syndrome score and global cognitive trajectory.

Importantly, for the moderate versus high trajectory comparison, the continuous analysis did not reveal a statistically significant association in the primary binary analysis. Each unit increase in CircS score was associated with a 7% increase in odds of belonging to the moderate versus high trajectory (adjusted OR = 1.07, 95% CI: 1.03–1.11). This finding suggests that the binary classification of CircS may have limited sensitivity to detect more subtle associations with intermediate cognitive outcomes, and that circadian dysregulation operates along a continuum in its relationship with cognitive trajectories (Figure 5).

#### 3.6.4 Sensitivity analysis with additional covariates

After adjusting for additional covariates, the association between CircS and the “persistently low” global cognitive trajectory remained significant, with an adjusted odds ratio of 1.31 (95% CI: 1.09–1.57; Table S4). In the causal forest analysis, the ATE of CircS on the “persistently low” trajectory was 0.0312 (95% CI: 0.0111–0.0513) [Table S5]. The distributions of ITEs are detailed in Table S5 and Figure S14. Variable importance analysis (Figure S14) identified Hb, BMI, and age as the top three moderators for the “persistently low” trajectory, consistent with the primary analysis. Physical activity emerged as the fourth most important variable. The relationships of predicted ITEs with Hb, BMI, and age in “persistently low” trajectory (Figure S15) remained similar to those in the primary analysis. A weak positive correlation was observed between physical activity and the ITEs in in “persistently low” trajectory (*r* = 0.09).

#### 3.6.5 Sensitivity analysis using multiple imputation

To evaluate the potential influence of selection bias due to missing baseline CircS data, analyses were repeated using multiple imputation. The trajectory patterns identified in the imputed data were similar to the primary analysis (Figure S16). In the adjusted multinomial logistic regression model, CircS remained associated with the “persistently low” global cognitive trajectory (adjusted OR = 1.13, 95% CI: 1.01–1.26; Table S6). Results from the causal forest analysis on the imputed data are summarized in Table S7. The ATE of CircS on the “persistently low” trajectory was 0.0209 (95% CI: 0.0096–0.0322).

Variable importance analysis (Figure S17) indicated that age, Hb, and BMI remained the three most influential moderators of the CircS effect across trajectories. The relationships between predicted ITEs and Hb and BMI in “persistently low” global cognitive trajectory (Figure S18) were consistent with the primary findings. However, the relationship with age differed, exhibiting a U‐shaped pattern in the imputed data, with higher treatment effects observed in both younger and older individuals.

#### 3.6.6 Sensitivity analysis examining collinearity and a modified CircS definition

We conducted two additional sensitivity analyses to examine the robustness of our findings regarding BMI as an effect moderator. First, VIFs for all covariates, including CircS and BMI, were calculated. All VIFs were below 5, indicating no substantial multicollinearity (Figure S19).

Second, we repeated the causal forest analysis using a modified definition of CircS that excluded the central obesity component. CircS was redefined as having ≥3 of the remaining 6 components. The association between this modified CircS and the “persistently low” cognitive trajectory remained significant, with an ATE of 0.0336 (95% CI: 0.0147–0.0525). Hemoglobin, age, and BMI were again identified as the top three moderators of this effect (Figures S20,21), and their moderating patterns were basically consistent with the primary analysis.

### 3.7 Extended analysis

In the extended analysis of cognitive transitions, the association between CircS and cognitive decline yielded an adjusted odds ratio of 1.04 (95% CI: 0.92–1.17; Table S8). The causal forest analysis yielded an ATE of 0.0010 (95% CI: −0.0273 to 0.0294). Substantial heterogeneity in ITEs was observed (SD = 0.0397; range: −0.0994 to 0.169; Figure S22). Variable importance analysis identified BMI, hemoglobin, and age as the three most influential moderators, and their effect modification patterns (Figure S23) were similar to those observed for the “persistently low” trajectory in the primary analysis.

## 4 DISCUSSION

This longitudinal study demonstrates that CircS is significantly associated with unfavorable cognitive trajectories among middle‐aged and older Chinese adults. After multivariable adjustment, individuals with CircS had a 27% higher risk of a “persistently low” global cognitive trajectory and a 28% higher risk of a “persistently low” episodic memory trajectory. Importantly, by utilizing LCGM, we showed that CircS predicts distinct long‐term cognitive aging patterns, moving beyond prior cross‐sectional evdience.^13^ These findings were consistent in the sensitivity analyses, the latter confirming accelerated decline in global cognitive function among individuals with CircS. Additionally, causal forest analysis revealed substantial heterogeneity in the effect of CircS, moderated primarily by hemoglobin levels, age, and BMI. Collectively, our findings establish CircS as a comprehensive and potent risk factor for cognitive decline.

### 4.1 Mechanisms between CircS and cognitive trajectory deterioration

1. Pathophysiological mechanism:

The pathway through which CircS contributes to cognitive decline initiates with the dysregulation of clock genes, such as BMAL1 and PER.^30^, ^31^, ^32^ This disruption directly compromises neuronal plasticity and synaptic functionality. Subsequently, it induces the activation of microglial cells, which release pro‐inflammatory mediators, including IL‐6 and TNF‐α, and results in the accumulation of mitochondrial reactive oxygen species (ROS).^33^, ^34^ Concurrently, metabolic and cerebrovascular dysfunctions impair cerebral blood flow perfusion and compromise the integrity of the blood–brain barrier, culminating in white matter lesions and microinfarcts.^35^ Additionally, abnormal melatonin secretion diminishes its neuroprotective effects against oxidative stress and Aβ aggregation.^36^, ^37^ Ultimately, sleep disturbances hinder the glymphatic system's ability to clear metabolic waste products, such as Aβ and tau, leading to the accumulation of neurodegenerative pathologies.^38^, ^39^ These processes are intricately linked through CircS disruptions and collectively exacerbate cognitive decline.

1. Psychological and Behavioral Mechanisms:

In addition to the biological pathways, the link between CircS and adverse cognitive trajectories may also involve behavioral and psychological pathways. Depressive symptoms may serve as a crucial mediator. Depression is frequently associated with apathy, diminished motivation, and anhedonia, which can lead to decreased participation in cognitively stimulating activities and social interactions—critical components of cognitive reserve.^40^, ^41^, ^42^

### 4.2 Heterogeneity analysis

Causal forest analysis demonstrated significant heterogeneity in the influence of CircS on cognitive trajectories, identifying hemoglobin, age, and BMI as key moderators. Unlike conventional regression approaches, our machine learning method quantified individual‐level variations and identified a high‐treatment‐effect subgroup: younger individuals with mild‐anemia hemoglobin levels (approximately 95 g/L) and elevated BMI.

Hemoglobin emerged as the most influential moderator, exhibiting a distinct nonlinear relationship with the effects of CircS. Rather than a simple linear association, a U‐shaped or threshold effect was observed: the most pronounced adverse effects of CircS were concentrated among individuals within an intermediate range of hemoglobin concentration (approximately 95 g/L), while those at both lower and higher extremes demonstrated relative resilience. This complex pattern indicates the presence of distinct underlying pathophysiology. Low hemoglobin may contribute to cognitive impairment primarily through chronic cerebral hypoxia^43^, ^44^, ^45^, ^46^, ^47^—a parallel risk pathway—whereas the concentration of risk in the middle range may signify a “metabolically‐vulnerable” phenotype. In these individuals, subclinical vascular dysfunction and low‐grade inflammation, core components of CircS, likely converge to induce cognitive damage without being severe enough to manifest as overt anemia.

Age emerged as the second most significant moderator, exhibiting an unexpected direction of effect. Our analysis revealed a negative correlation between age and the ITE of CircS, suggesting that the adverse impact on cognitive decline was more pronounced among younger individuals. This observation may imply the presence of a critical period in mid‐life during which the intersection of circadian, metabolic, and affective disturbances represented by CircS could trigger a more rapid and potentially less reversible pathological progression, thereby establishing a trajectory for accelerated long‐term decline.^9^, ^48^, ^49^, ^50^, ^51^, ^52^ Alternatively, this pattern could reflect a selective survival bias, wherein the oldest‐old participants who remain in the study are inherently more resilient.

BMI was also a significant moderator, a finding that requires careful interpretation. It is important to distinguish the role of BMI as a moderator from central obesity as a diagnostic component of CircS. While central obesity specifically reflects abdominal visceral fat, a key driver of cardiometabolic risk within CircS, BMI serves as a broader, composite indicator of overall body composition and metabolic load. It captures not only adiposity but also aspects of nutritional status and chronic, low‐grade systemic inflammation—factors implicated in neuroinflammation and cognitive decline through pathways distinct from, though often concomitant with, abdominal fat accumulation.^53^, ^54^, ^55^, ^56^ Therefore, BMI may represent a dimension of generalized metabolic vulnerability that extends beyond the specific criterion of central obesity. The observed moderating effect of BMI likely operates through these extended pathways, which may interact synergistically with circadian dysfunction to exacerbate cognitive decline. For instance, elevated BMI frequently coincides with a state of “metabolically unhealthy obesity,” characterized by insulin resistance and altered adipokine secretion—conditions that are themselves both causes and consequences of circadian disruption.^57^, ^58^, ^59^, ^60^ This bidirectional relationship suggests a vicious cycle wherein circadian misalignment and metabolic dysfunction mutually reinforce each other, creating a compounded risk for neural integrity. In this context, BMI emerges not merely as a correlate of abdominal obesity but as a clinically accessible marker of this integrated, pathological network. Thus, its role as an effect modifier likely reflects a distinct layer of metabolic susceptibility that influences an individual's resilience or vulnerability to the cognitive impacts of CircS.

### 4.3 Clinical implications

Our findings have significant implications for clinical practice and public health initiatives aimed at preserving cognitive function. This longitudinal study demonstrates that CircS predicts the “persistently low” cognitive trajectory. Furthermore, a dose–response relationship was observed, wherein a higher CircS score increased the risk of belonging to the persistently low cognitive trajectory. The identification of CircS as a strong predictor of adverse cognitive outcomes highlights its potential as a clinically actionable screening tool. In primary care and geriatric settings, evaluating the composite CircS profile could enable the early identification of middle‐aged and older adults at elevated risk for persistent cognitive decline, thereby facilitating timely interventions before substantial neurodegeneration occurs. Moreover, the heterogeneity revealed by our causal forest analysis strongly supports a stratified and personalized prevention approach. Specifically, individuals with mild anemia (hemoglobin levels around 95 g/L) and those in the younger middle‐age bracket emerge as priority subgroups who may benefit most from targeted management of their circadian and metabolic health. Potential interventions may encompass lifestyle modifications aimed at aligning circadian rhythms, such as scheduled light exposure and consistent meal and sleep routines, as well as strategies for metabolic enhancement. Future research ought to prioritize the development and rigorous evaluation of these multifaceted interventions, with a particular emphasis on conducting randomized controlled trials. Such studies are essential to ascertain whether addressing components of CircS factors can effectively slow cognitive decline in identified high‐risk populations.

### 4.4 Strength and limitation

This study possesses several methodological strengths. First, the utilization of a large, nationally representative cohort with four waves of longitudinal data allowed us to move beyond cross‐sectional snapshots and model the dynamic nature of cognitive change, providing a more robust assessment of the exposure–outcome relationship.^61^, ^62^ Second, our application of LCGM was pivotal in capturing the inherent heterogeneity in cognitive aging, identifying distinct subpopulations with “persistently low,” “moderate,” and “high” trajectories that would be obscured by analyzing the population average alone.^63^, ^64^ Third, we complemented this group‐based trajectory analysis with a cutting‐edge machine learning technique—causal forest analysis—to estimate ITEs. This advanced method allowed us to quantify the substantial heterogeneity in how CircS affects cognitive trajectories and to identify key moderators and a high‐risk subgroup, thereby providing a foundation for personalized risk assessment.^65^, ^66^

Several limitations should be considered when interpreting our findings. First, the observational design inherently limits causal inference, despite robust sensitivity analyses. Factors like social engagement may have bidirectional relationships with CircS, complicating full adjustment. Furthermore, the lack of objective measures for conditions like sleep apnea in CHARLS means residual confounding cannot be ruled out. Thus, our findings, while robust, cannot fully satisfy the assumption of no unmeasured confounding. Second, the assessment of key CircS components, particularly sleep and depressive symptoms, relied on self‐reports, which may introduce measurement bias. This limitation is especially pertinent for the sleep component. Our definition used sleep duration alone (<6 h), a metric that does not capture other core dimensions of sleep health (e.g., regularity, timing).^27^ Since short sleep can arise from diverse causes (e.g., behavior, natural phenotype) not specific to circadian dysfunction,^67^, ^68^, ^69^ our measure may lead to misclassification and potentially underestimate the true association between circadian disruption and cognitive trajectories. Future studies should employ multidimensional sleep assessments or circadian biomarkers to better define this component of the syndrome. Third, the generalizability of our findings may be limited to middle‐aged and older Chinese adults, and replication in other ethnic and younger populations is needed. Finally, the conceptual overlap between BMI and the central obesity component of CircS complicates the interpretation of BMI as a distinct moderator. Although sensitivity analyses indicated that BMI's moderating role persists even when central obesity is excluded from the syndrome definition, BMI likely reflects correlated metabolic features of the exposure.

## 5 CONCLUSION

This study illustrates CircS significantly elevates the risk of adverse cognitive trajectories among Chinese middle‐aged and older adults. Individuals with CircS exhibited a 27% increased risk of persistent global cognitive decline and a 28% increased risk of poor episodic memory trajectory. Causal forest analysis revealed considerable heterogeneity in individual susceptibility, identifying a high‐treatment‐effect subgroup characterized by younger age, mid‐anemia hemoglobin levels, and elevated BMI. These findings establish CircS as a significant and modifiable risk factor for cognitive trajectory deterioration. Incorporating circadian health assessments into clinical practice could facilitate the early identification of at‐risk individuals, particularly those exhibiting the identified risk profile. Future research should prioritize the development of targeted interventions that address circadian disruption and its metabolic correlates to mitigate cognitive consequences in vulnerable populations.

## AUTHOR CONTRIBUTIONS

**Jiangping Ma**: Study design, data collection, formal analysis, visualization, and writing an original draft. **Nan Chen**: Study design, data collection, formal analysis, visualization, and writing an original draft. **Jingxuan Huang**: Data collection, formal analysis, and writing an original draft. **Linghao Xu**: Writing an original draft. **Huifang Shang**: Supervision, project administration, writing a review & editing. All authors read and approved the final manuscript.

## FUNDING INFORMATION

This work was supported by the Sichuan Science and Technology Program (Grant No. 2022ZDZX0023).

## CONFLICT OF INTEREST STATEMENT

The authors declare no conflicts of interest. Author disclosures are available in the supporting information.

## ETHICAL APPROVAL

The protocol received approval from the Ethical Review Committee of Peking University (approval numbers: IRB00001052‐11015 for the main household survey and IRB00001052‐11014 for biomarker collection). The study was conducted in compliance with the Declaration of Helsinki principles. All participants signed the informed consent and repository consent that permitted their data to be shared after a detailed presentation of the risks and benefits related to study participation.

## Supporting information



Supporting Information

Supporting Information

Supporting Information

## Data Availability

The datasets utilized in this investigation are available in online repositories. The names of the repository and the accession number(s) can be found at the following link: http://charls.pku.edu.cn/en.
