# Supplementary material for: Circadian syndrome (CircS) and cognitive trajectory deterioration in middle‐aged and older adults: A national cohort study with causal forest analysis
Source: Alzheimers Dement. 2026 Mar 18;22(3):e71300. doi: 10.1002/alz.71300 (PMC13093444; doi:10.1002/alz.71300)
Supplement: Supplementary file 1 — Supporting Information [file ALZ-22-e71300-s003.docx]

·
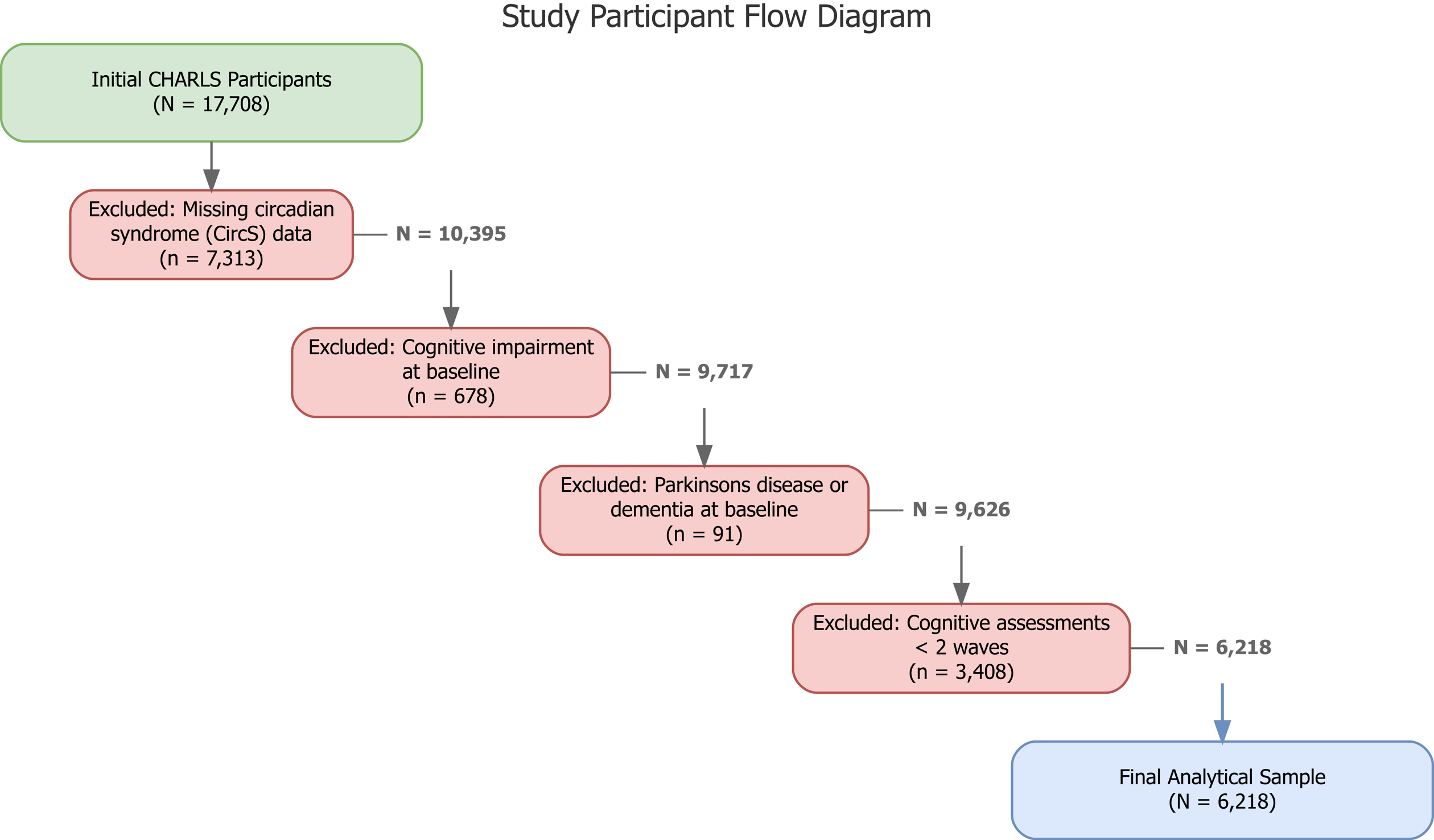


**Figure S1** Inclusion and Exclusion Flowchart.





**Figure S2** Age-specific corresponding cognitive impairment thresholds (mean - 1.5 SD)


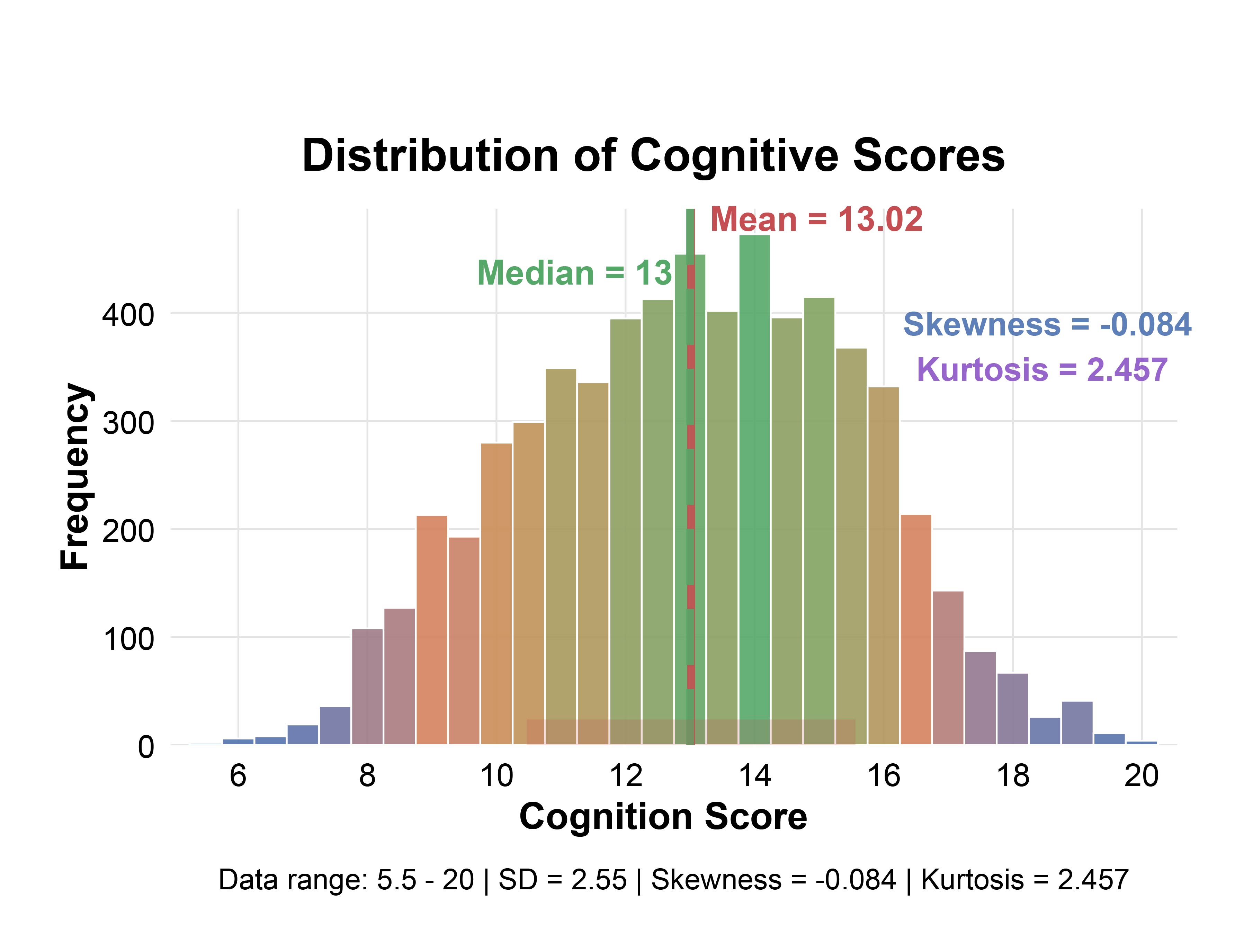


**Figure S3** Distribution of Global Cognitive Scores at Baseline

**
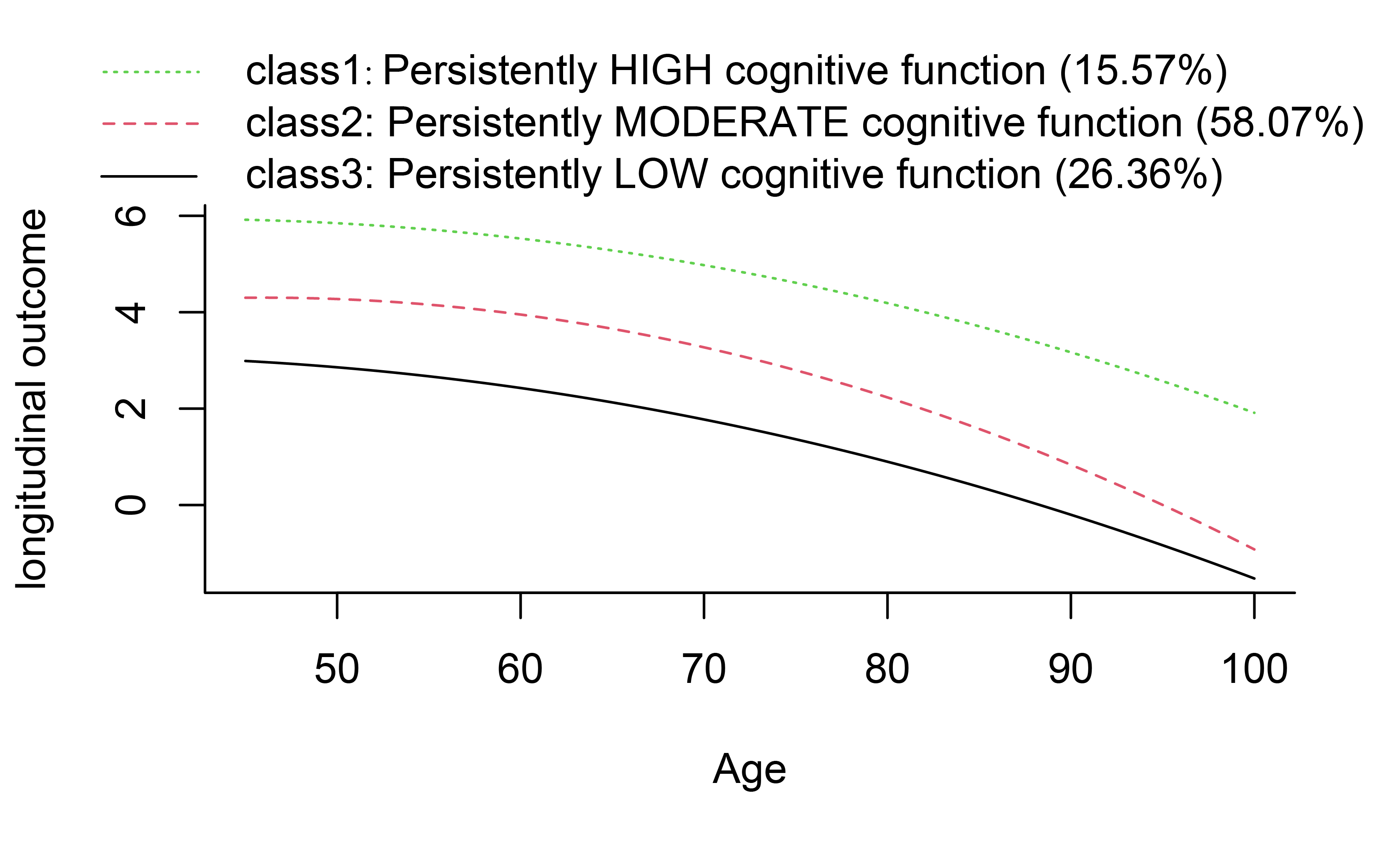

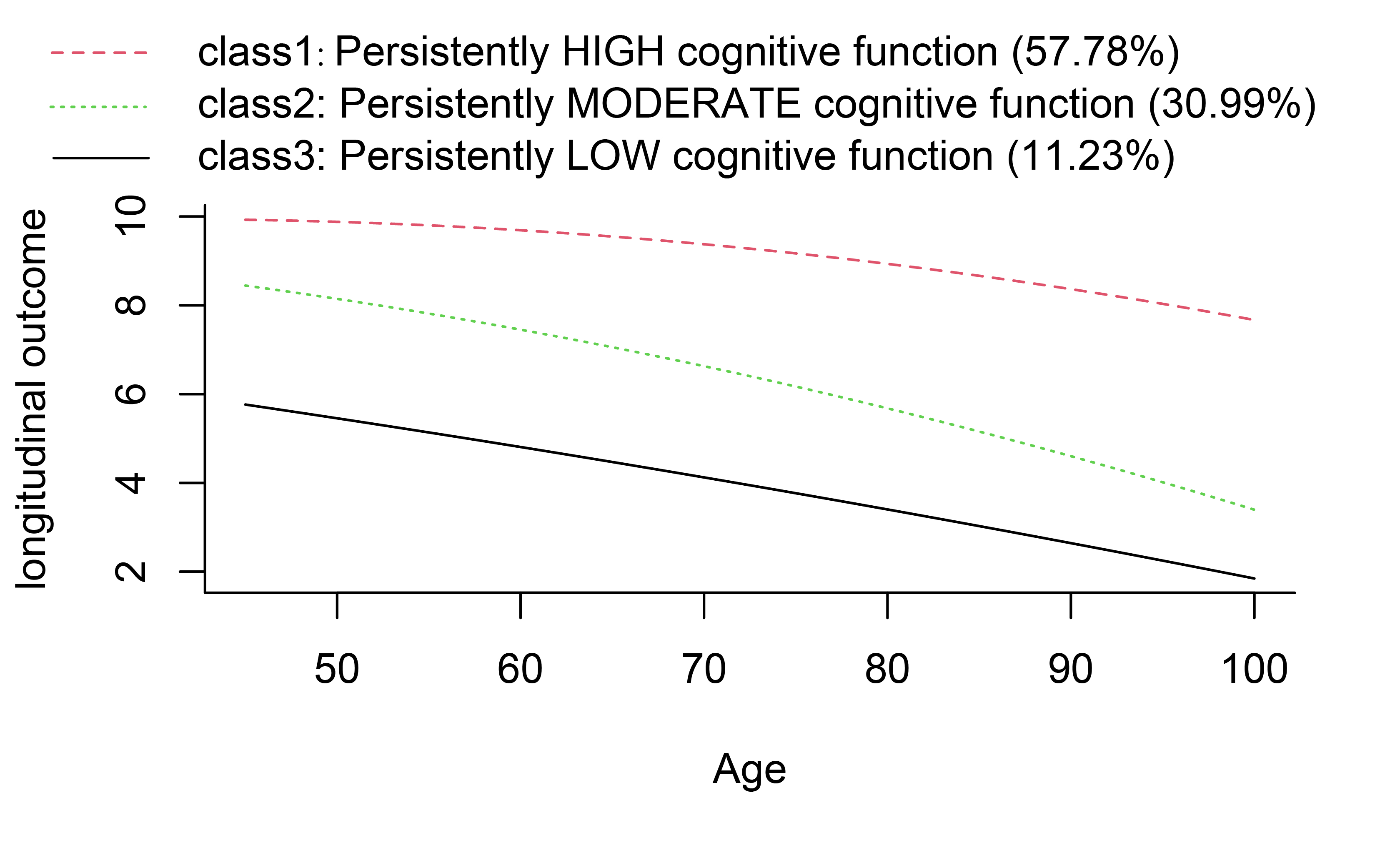
**

**Figure S4** Mean trajectories of episodic memory scores (left) and mental intactness scores (right) by increasing age among older adults


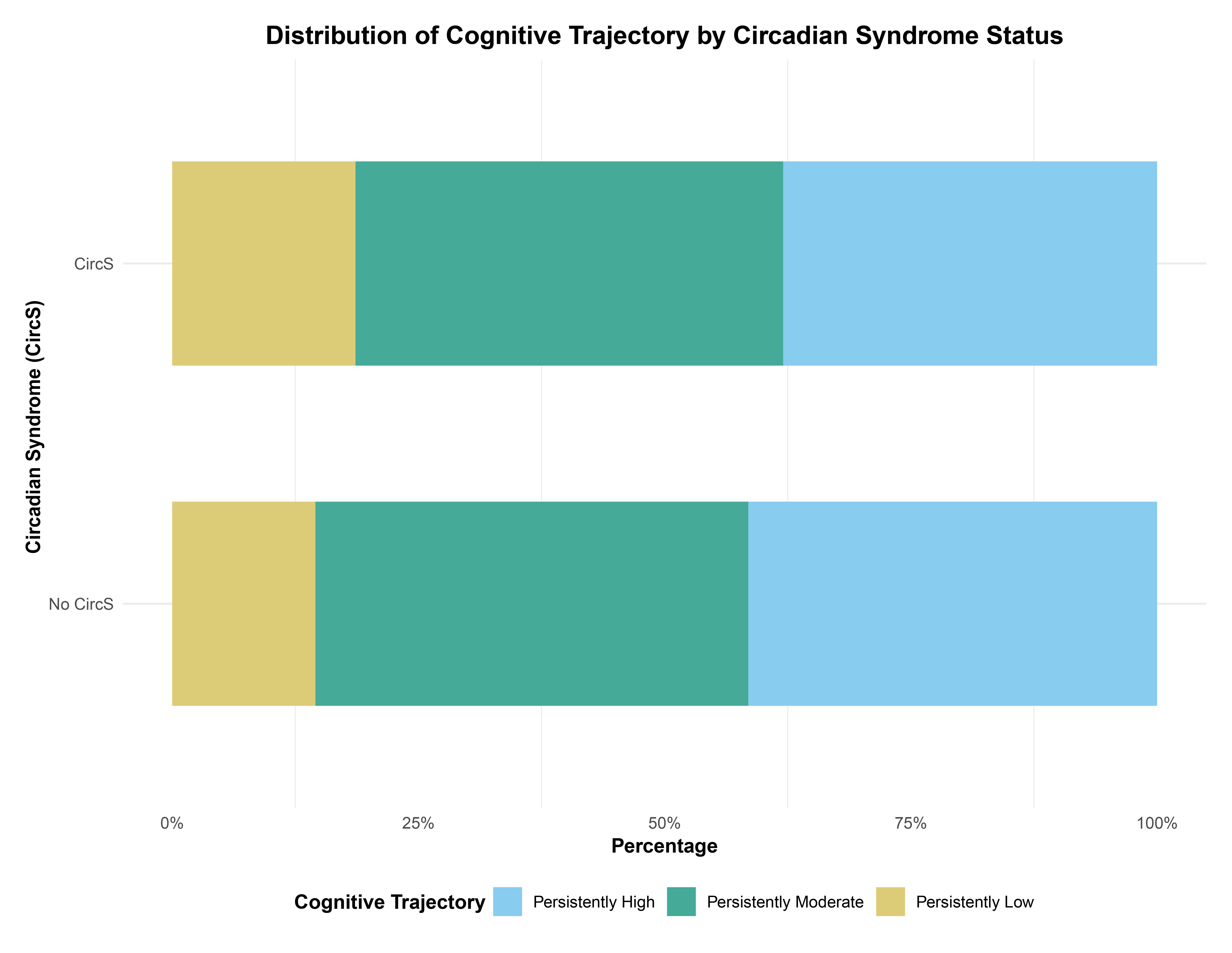


**Figure S5** Distribution of Global Cognitive Trajectories by Circadian syndrome (CircS) Status

[Note: This bar chart illustrates the distribution of participants across "High," "Moderate," and "Low" cognitive trajectories, stratified by the presence or absence of CircS. A chi-square test indicated a significant association between CircS status and cognitive trajectory distribution (P < 0.001)].





**Figure S6** Stratified analysis of the association between circadian syndrome and episodic memory trajectory membership across all covariates, with interaction p-values





**Figure S7** Stratified analysis of the association between circadian syndrome and mental intactness trajectory membership across all covariates, with interaction p-values


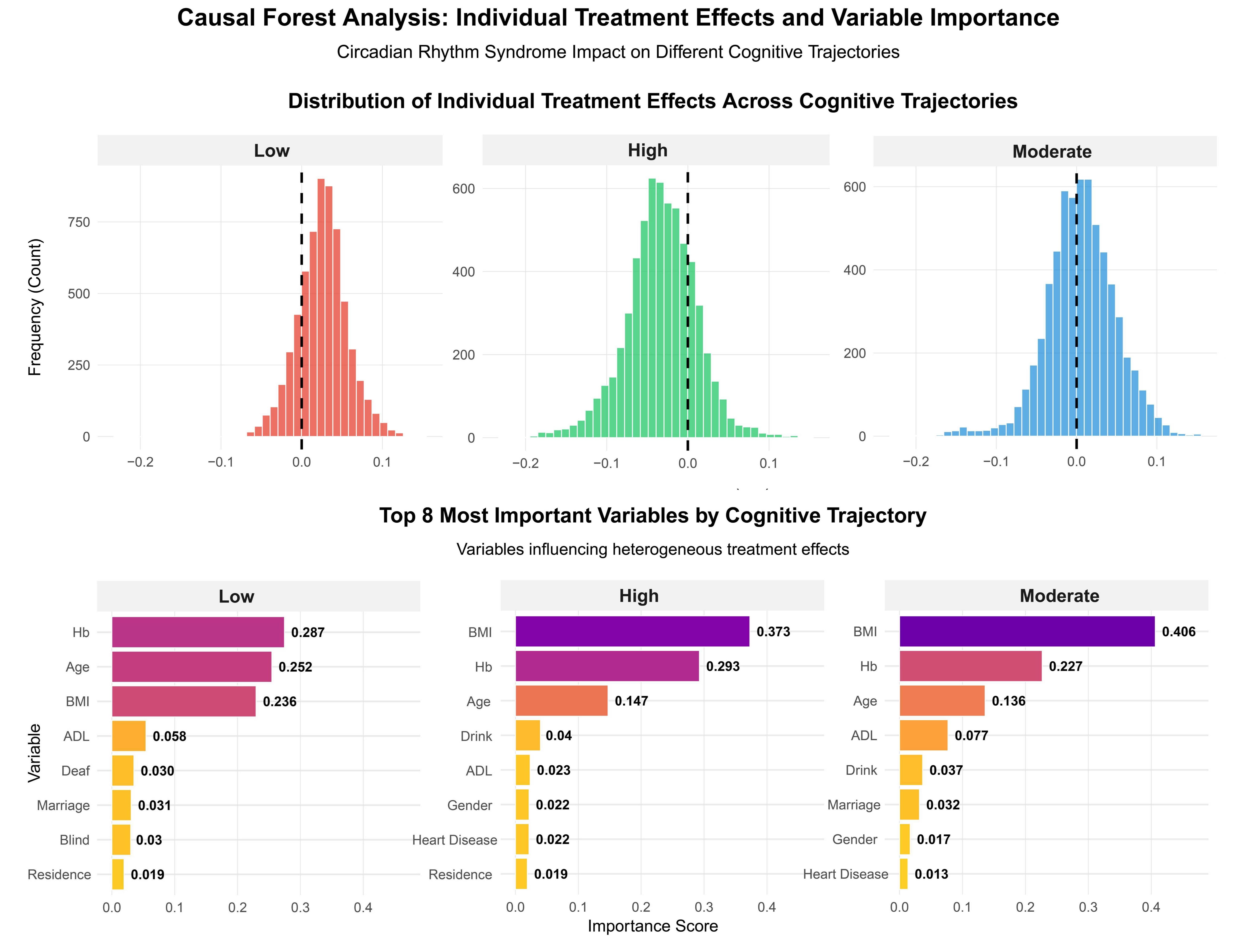


**Figure S8** Distribution of individual treat effects and variable importance across different cognitive trajectories


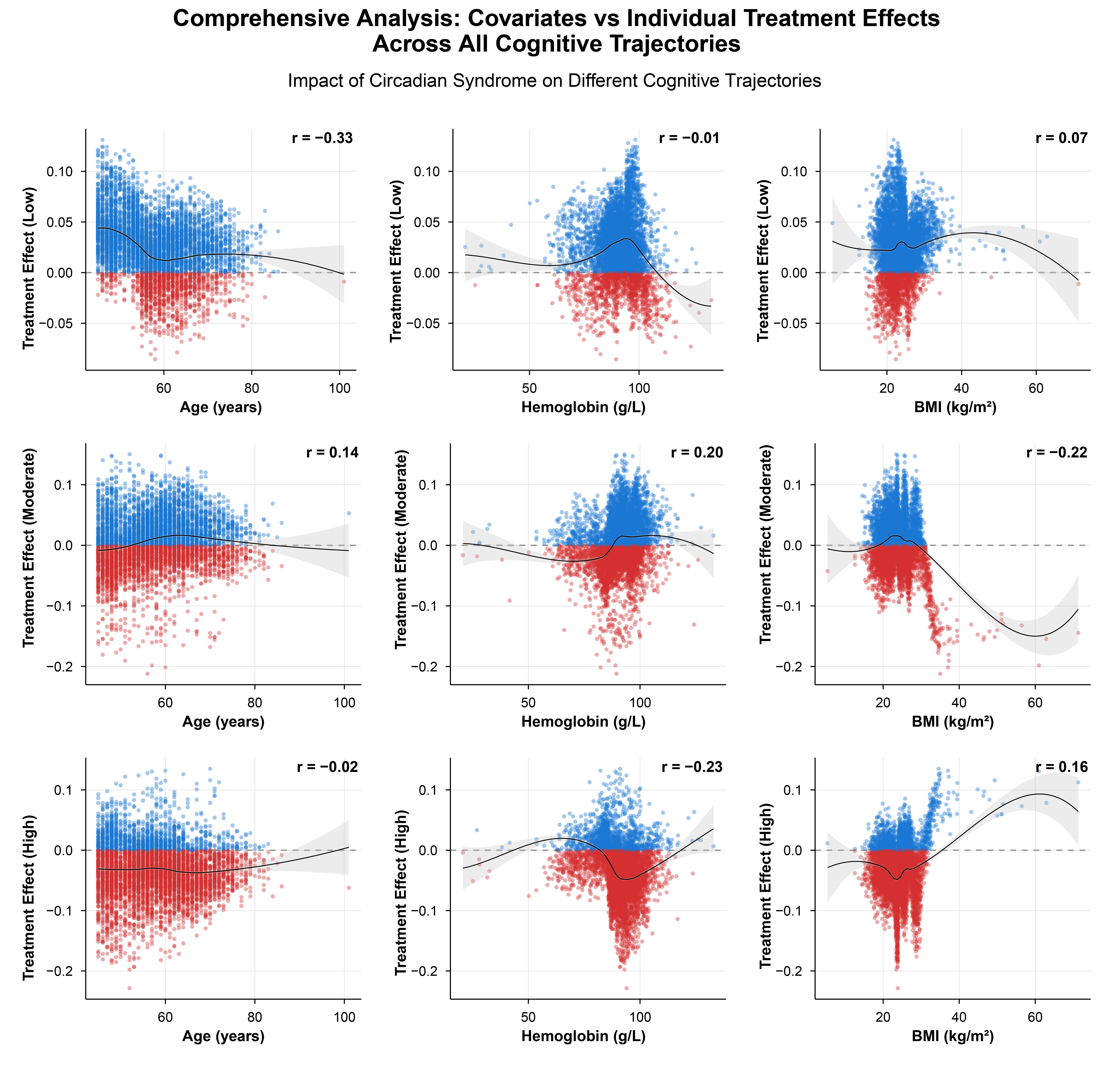


**Figure S9** The predictive impact of circadian syndrome on three different cognitive trajectories and its relationship with age, hemoglobin level, and body mass index [persistently low (top panel), persistently moderate (middle panel), and persistently high (bottom panel)].





**Figure S10** Covariate Balance Before and After Inverse Probability Weighting





**Figure S11** Distribution of Inverse Probability Weights by Circadian Syndrome Status





**Figure S12** Distribution of Cognitive Trajectories Before and After Inverse Probability Weighting


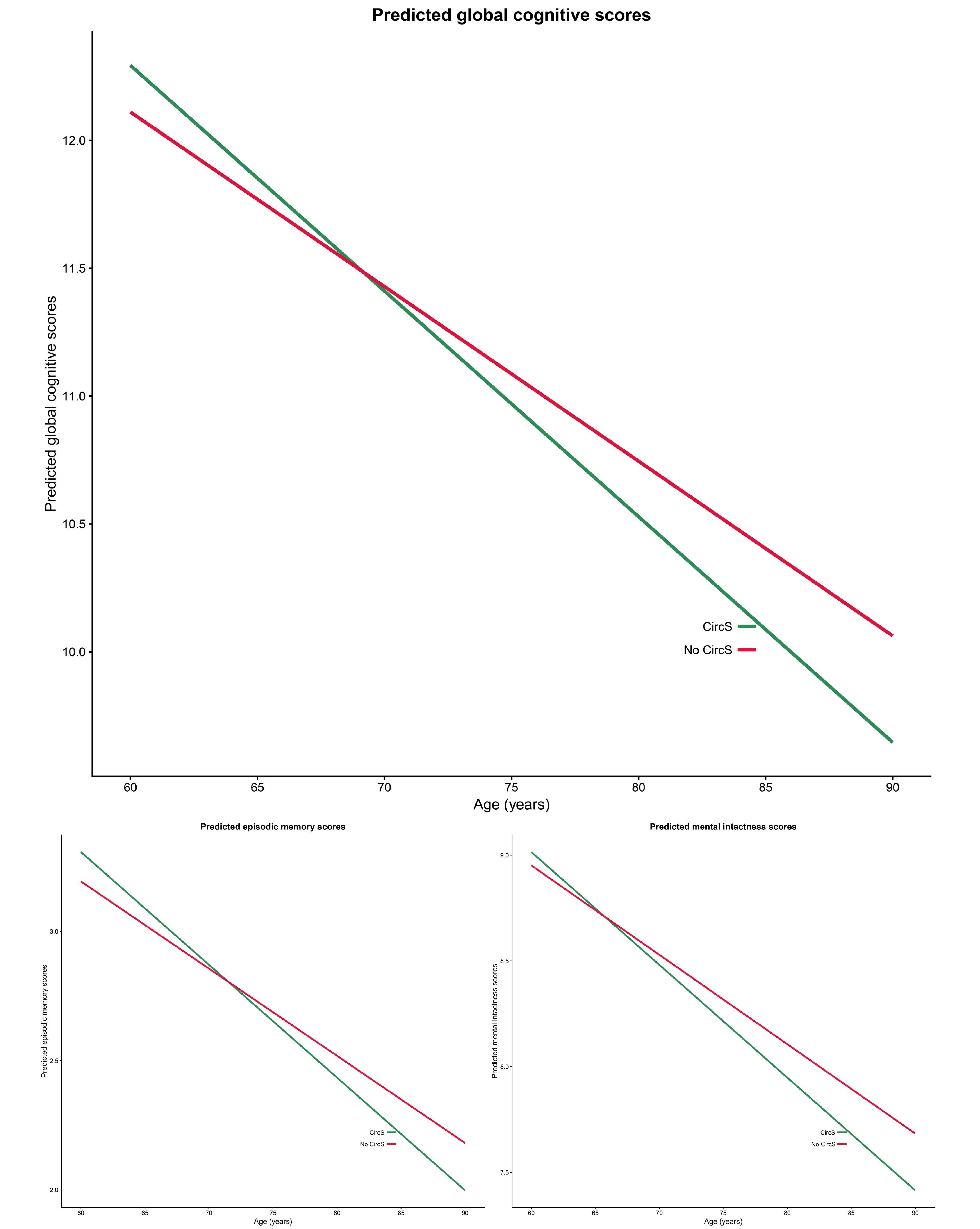


**Figure S****13** Predicted Trajectories of Cognitive Function by Age and Circadian syndrome (CircS) Status from Linear Mixed-Effects Models.


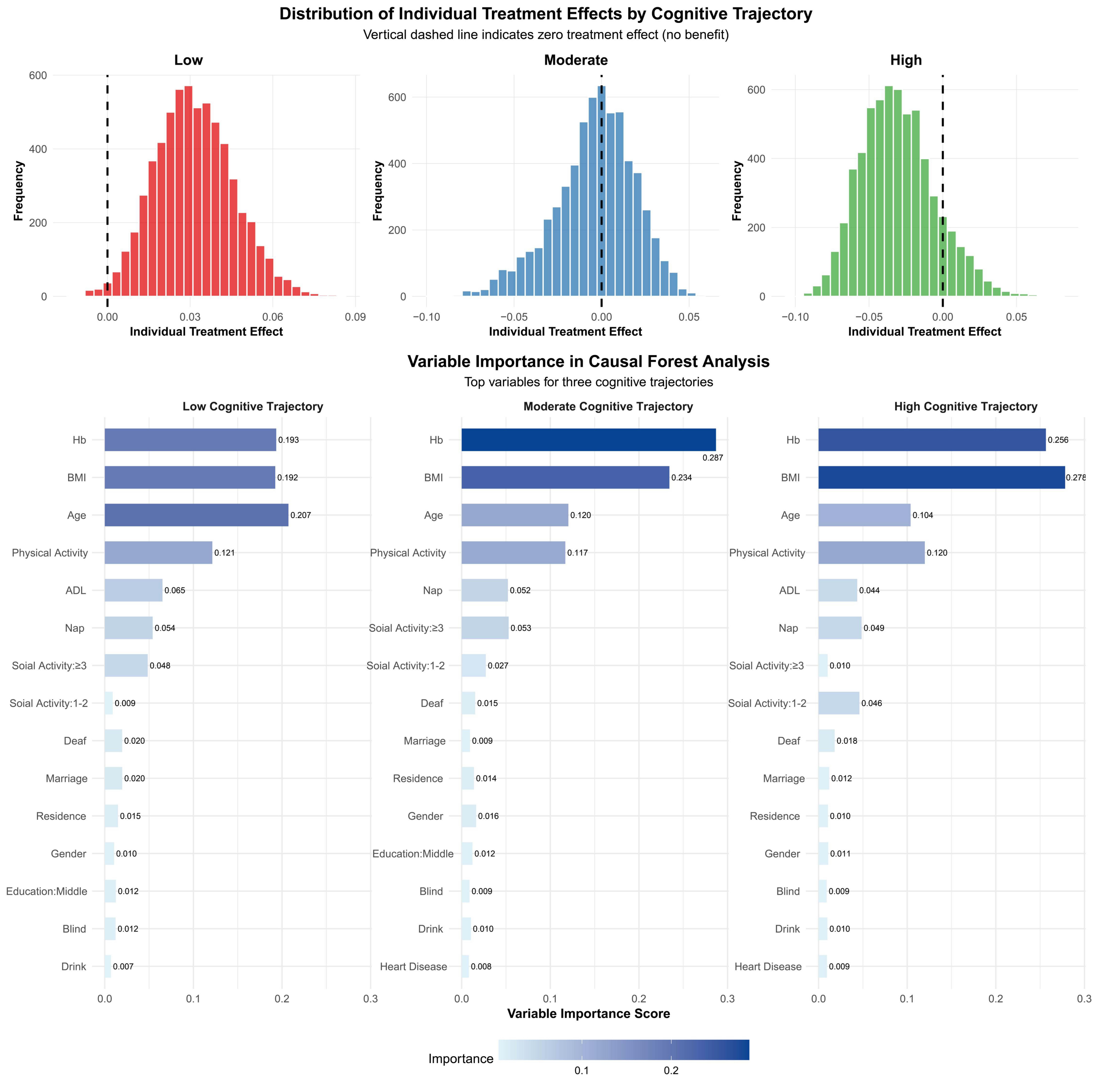


**Figure S14** Distribution of individual treat effects and variable importance across different cognitive trajectories (Sensitivity Analysis with Additional Covariates)


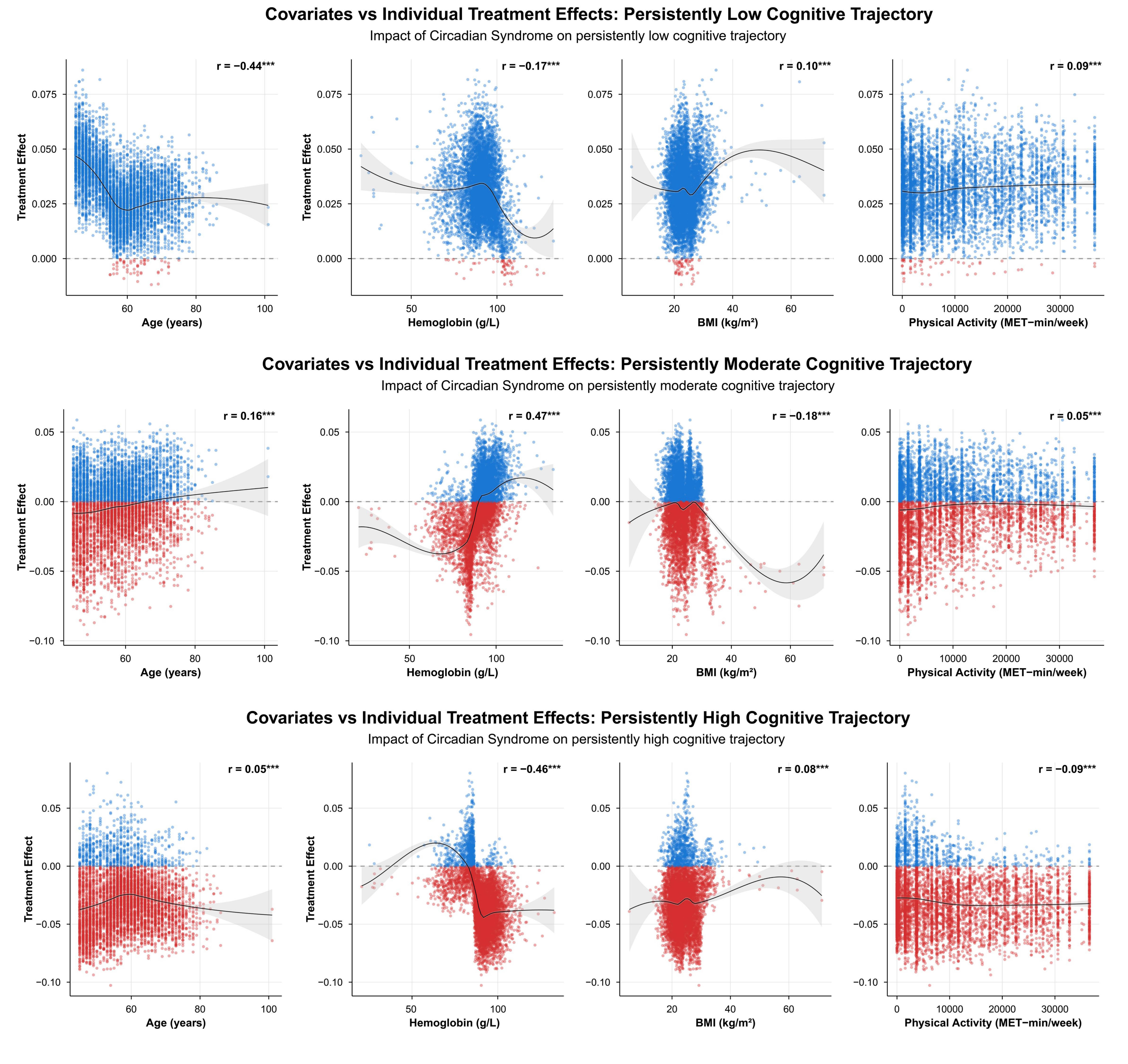


**Figure S15** The predictive impact of circadian syndrome on three different cognitive trajectories and its relationship with age, hemoglobin level, body mass index, and physical activity [Sensitivity Analysis with Additional Covariates].


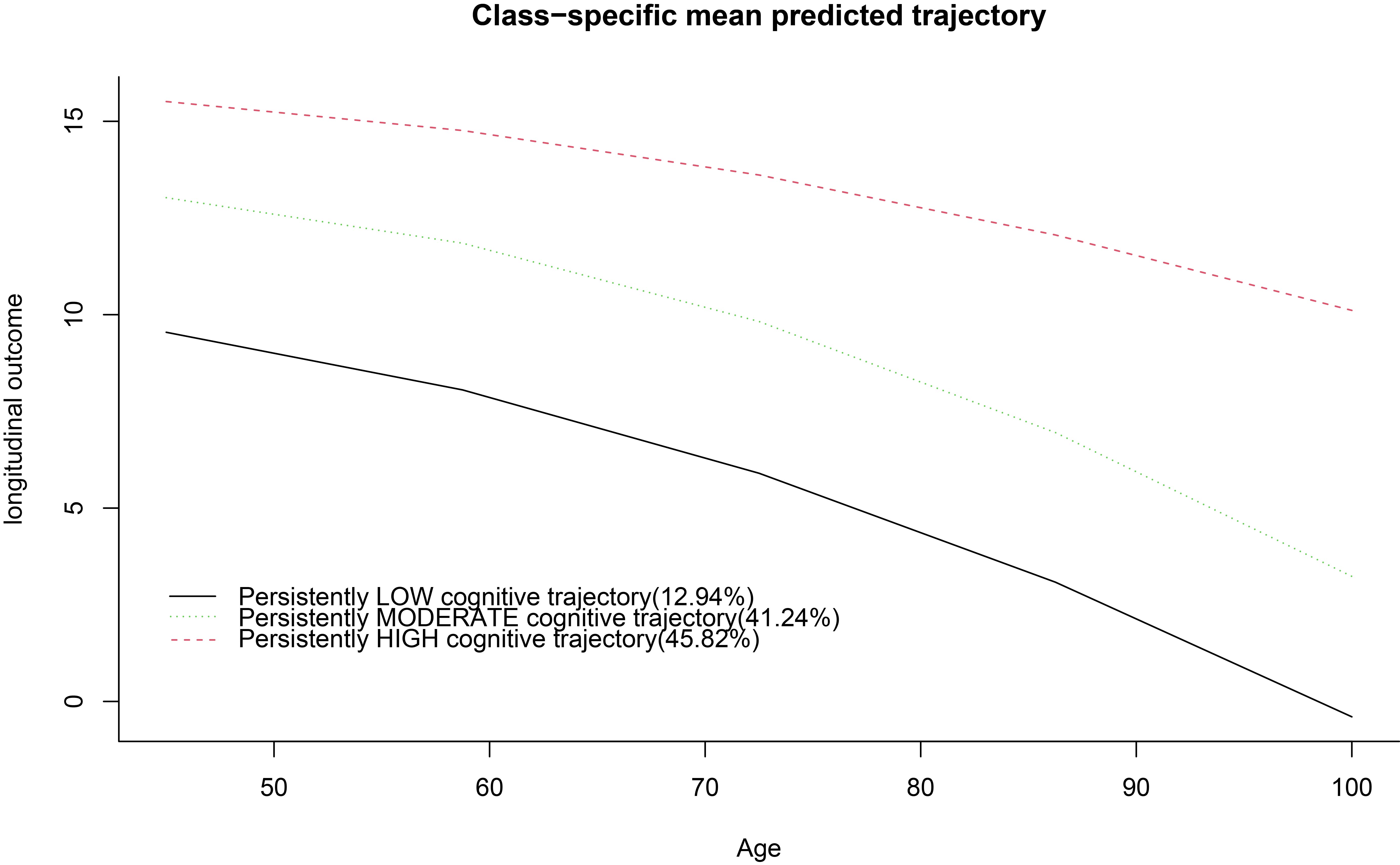


**Figure S16** Mean trajectories of global cognitive scores by increasing age among participants (Multiple Imputation on missing CircS)

**

**

**Figure S17** Distribution of individual treat effects and variable importance across different cognitive trajectories (Multiple Imputation on missing CircS)

**

**

**Figure S18** The predictive impact of circadian syndrome on three different cognitive trajectories and its relationship with age, hemoglobin level, and body mass index (Multiple Imputation on missing CircS).





**Figure S19** The variance inflation factor between CircS and the covariates

**

**

**Figure S20** Causal Forest Analysis of Circadian syndrome Effects on Cognitive Trajectories (with a Modified CircS Definition)

**
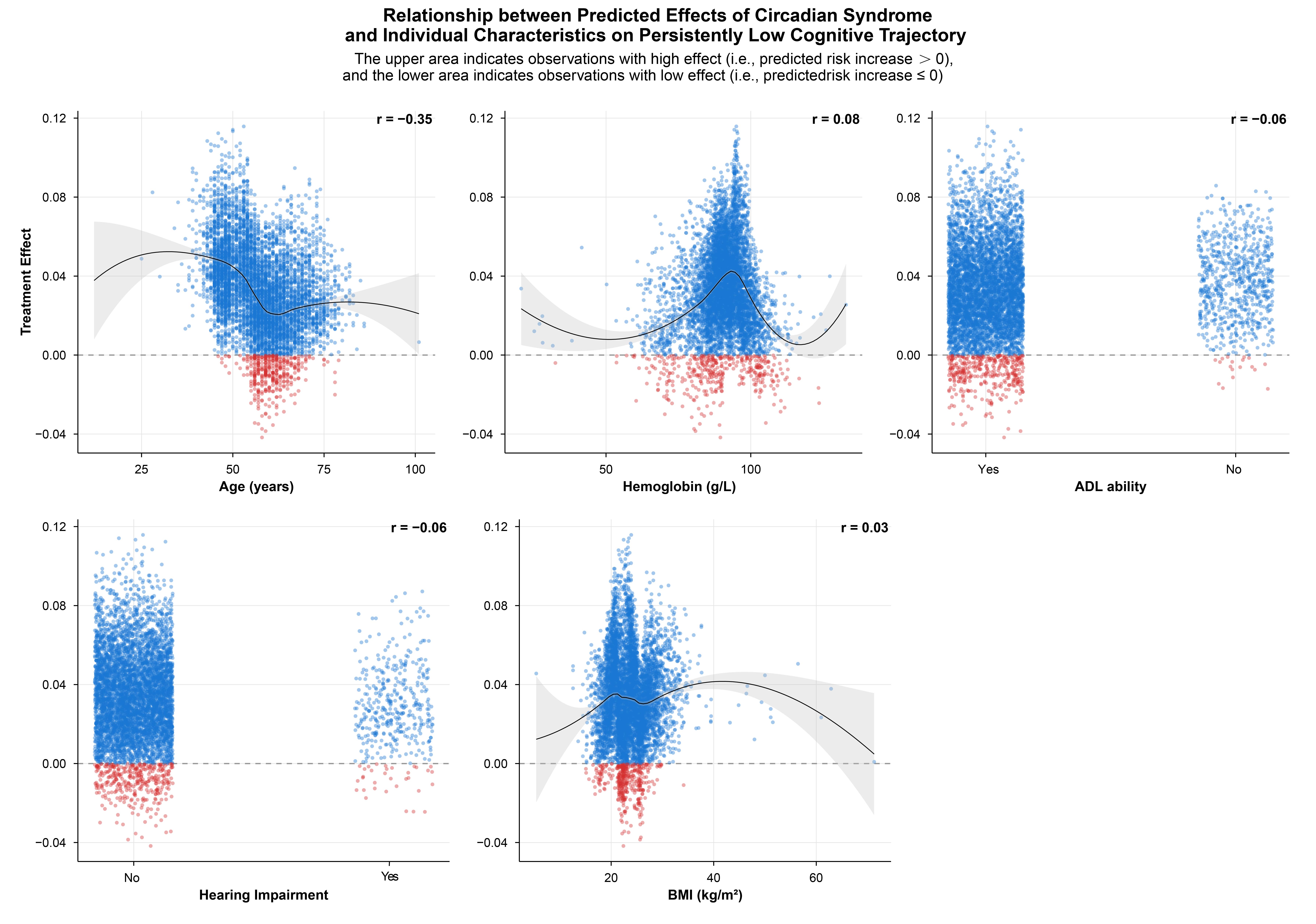
**

**Figure S21** The predictive impact of circadian syndrome (with a Modified CircS Definition) on persistently low cognitive trajectories and its relationship with age, hemoglobin level, ADL ability, hearing impairment, and body mass index.





**Figure S22** Causal Forest Analysis of Circadian syndrome Effects on Cognitive Transition


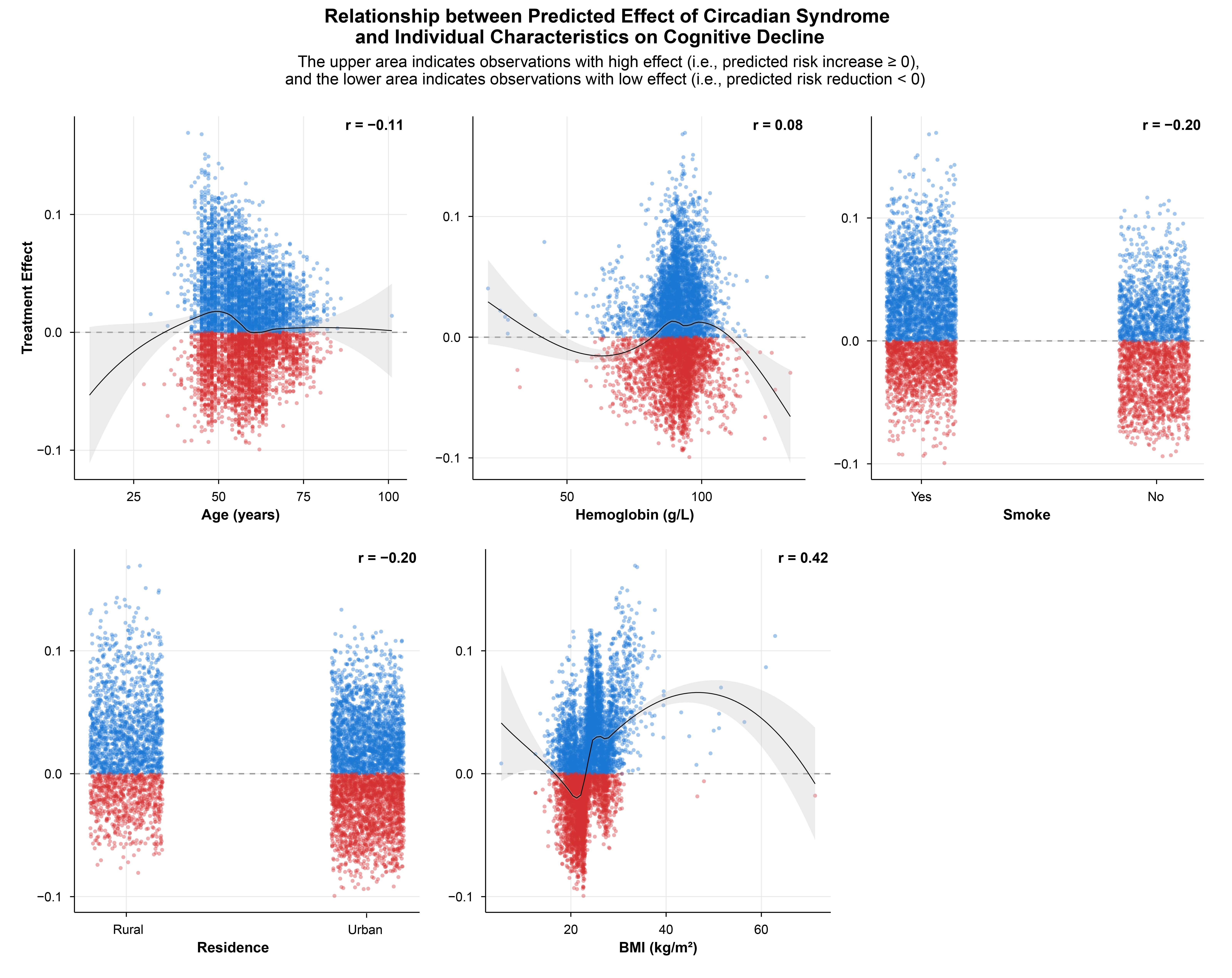


**Figure S23** The predictive impact of circadian syndrome on Cognitive Transition and its relationship with age, hemoglobin level, smoking status, residence, and body mass index.

**Table S1** Fit statistics for global cognitive function group trajectories in middle-aged and older adults from CHARLS

| Fit statistic | Number of classes | | | | |
| --- | --- | --- | --- | --- | --- |
|  | 1 | 2 | 3 | 4 | 5 |
| AIC* | 105070.71 | 98992.07 | 97197.26 | 96778.5 | 96592.37 |
| BIC* | 105097.65 | 99045.95 | 97278.08 | 96886.26 | 96727.08 |
| SABIC* | 105084.94 | 99020.53 | 97239.95 | 96835.42 | 96663.52 |
| entropy^‡^ | 1 | 0.7741445 | 0.7363401 | 0.6830504 | 0.6749396 |
| smallest class (%) ^¶^ | 100 | 35.670634 | 15.937601 | 8.025088 | 3.522033 |

AIC Akaike’s information criterion, BIC Bayesian information criteria,

*A lower absolute value suggests a better model fit

^¶^No less than 5% of total count in a class

^‡^A higher value is better (preferably > 0.7 in a class)

**Table S2** Baseline characteristics of the participants according to trajectories of global cognitive function in middle-aged and older adults from CHARLS

| **Variable** | **All (N = 6218)** | **Persistently high** | **Persistently moderate** | **Persistently low** | | ***P*.overall** |  |
| --- | --- | --- | --- | --- | --- | --- | --- |
|  |  | **Cognitive Trajectories** | | | |  |  |
| Age (years), mean ± SD | 57.35 ± 8.65 | 57.04 ± 8.79 | 57.44 ± 8.62 | 57.88 ± 8.32 | | 0.0270 |  |
| Hb (g/L), mean ± SD | 90.58 ± 8.53 | 90.79 ± 8.08 | 90.41 ± 8.94 | 90.52 ± 8.47 | | 0.2570 |  |
| Gender, n (%) |  |  |  |  | |  |  |
| Male sex | 3054 (49.1%) | 1406 (56.1%) | 1320 (48.5%) | 328 (33.1%) | | <0.0001 |  |
| Female sex | 3164 (50.9%) | 1099 (43.9%) | 1402 (51.5%) | 663 (66.9%) | | |  |
| Residence, n (%) |  |  |  |  | |  |  |
| Urban | 2186 (35.2%) | 1092 (43.6%) | 863 (31.7%) | 231 (23.3%) | | <0.0001 |  |
| Rural | 4032 (64.8%) | 1413 (56.4%) | 1859 (68.3%) | 760 (76.7%) | | |  |
| Educational level, n (%) | |  |  |  | |  |  |
| Below primary school | 4137 (66.5%) | 1260 (50.3%) | 1969 (72.3%) | 908 (91.6%) | | <0.0001 |  |
| Secondary to vocational school | 2024 (32.6%) | 1200 (47.9%) | 744 (27.3%) | 80 (8.1%) | | |  |
| University and above | 57 (0.9%) | 45 (1.8%) | 9 (0.3%) | 3 (0.3%) | |  |  |
| Marital status, n (%) | |  |  |  | |  |  |
| Unmarried | 483 (7.8%) | 156 (6.2%) | 218 (8.0%) | 109 (11.0%) | | <0.0001 |  |
| Married | 5735 (92.2%) | 2349 (93.8%) | 2504 (92.0%) | 882 (89.0%) | | |  |
| Smoking status, n (%) | |  |  |  | |  |  |
| No | 3715 (59.7%) | 1420 (56.7%) | 1626 (59.7%) | 669 (67.5%) | | <0.0001 |  |
| Yes | 2503 (40.3%) | 1085 (43.3%) | 1096 (40.3%) | 322 (32.5%) | | |  |
| Drinking status, n (%) | |  |  |  | |  |  |
| No | 3729 (60.0%) | 1443 (57.6%) | 1631 (59.9%) | 655 (66.1%) | | <0.0001 |  |
| Yes | 2489 (40.0%) | 1062 (42.4%) | 1091 (40.1%) | 336 (33.9%) | | |  |
| Restriction on ADL, n (%) | |  |  |  | |  |  |
| No | 5453 (87.7%) | 2299 (91.8%) | 2373 (87.2%) | 781 (78.8%) | | <0.0001 |  |
| Yes | 765 (12.3%) | 206 (8.2%) | 349 (12.8%) | 210 (21.2%) | | |  |
| History of stroke, n (%) | |  |  |  | |  |  |
| No | 6104 (98.2%) | 2457 (98.1%) | 2676 (98.3%) | 971 (98.0%) | | 0.7428 |  |
| Yes | 114 (1.8%) | 48 (1.9%) | 46 (1.7%) | 20 (2.0%) | | |  |
| Heart disease, n (%) | |  |  |  | |  |  |
| Yes | 620 (10.0%) | 276 (11.0%) | 255 (9.4%) | 89 (9.0%) | | 0.0727 |  |
| No | 5598 (90.0%) | 2229 (89.0%) | 2467 (90.6%) | 902 (91.0%) | | |  |
| BMI (kg/m2), n (%) |  |  |  |  | |  |  |
| < 18.5 | 343 (5.5%) | 107 (4.3%) | 166 (6.1%) | 70 (7.1%) | | <0.0001 |  |
| 18.5-23.9 | 3235 (52.0%) | 1246 (49.7%) | 1455 (53.5%) | 534 (53.9%) | | |  |
| 24.0-27.9 | 1915 (30.8%) | 828 (33.1%) | 805 (29.6%) | 282 (28.5%) | | |  |
| ≥ 28.0 | 725 (11.7%) | 324 (12.9%) | 296 (10.9%) | 105 (10.6%) | | |  |
| Visual impairment, n (%) | |  |  |  | |  |  |
| No | 5876 (94.5%) | 2399 (95.8%) | 2569 (94.4%) | 908 (91.6%) | | <0.0001 |  |
| Yes | 342 (5.5%) | 106 (4.2%) | 153 (5.6%) | 83 (8.4%) | | |  |
| Hearing impairment, n (%) | |  |  |  | |  |  |
| No | 5796 (93.2%) | 2369 (94.6%) | 2543 (93.4%) | 884 (89.2%) | | <0.0001 |  |
| Yes | 422 (6.8%) | 136 (5.4%) | 179 (6.6%) | 107 (10.8%) | | |  |
| Hb (g/L), mean ± SD | 90.58 ± 8.53 | 90.79 ± 8.08 | 90.41 ± 8.94 | 90.52 ± 8.47 | 0.2570 | |  |

SD: standard deviation; BMI: body mass index; Hb: Hemoglobin; CircS: Circadian syndrome.

**Table S3** Multinomial logistic regression analysis for the associations of continuous CircS score with the membership to cognitive function trajectory group

|  | Persistently low (vs persistently high) | | Persistently moderate (vs persistently high) | |
| --- | --- | --- | --- | --- |
|  | OR (95% CI) * | | OR (95% CI) * | |
| Global cognitive scores |  |  |  |  |
| Crude model | 1.15[1.10-1.21] | | 1.05[1.02-1.09] | |
| Adjusted model* | 1.16[1.05-1.18] | | 1.07[1.03-1.11] | |
| Mental intactness scores |  |  |  |  |
| Crude model | 1.11[1.06-1.17] | | 1.08[1.05-1.12] | |
| Adjusted model* | 1.07[0.99-1.21] | | 1.08[0.97-1.23] | |
| Episodic memory scores |  |  |  |  |
| Crude model | 1.09[1.03-1.14] | | 1.04[0.99-1.09] | |
| Adjusted model* | 1.10[1.04-1.17] | | 1.05[0.99-1.11] | |

95%CI: 95% confidence interval;

*Adjusted for age at baseline (continuous), gender (male, female), education (primary school or below, junior high school to high school, undergraduate or above), marital status (married, unmarried), residence (urban, rural), smoking (yes, no), drinking (yes, no), body mass index (< 18.5, 18.5–23.9, 24.0–27.9, ≥ 28.0 kg/m2), restriction on activities of daily living (yes, no), visual impairment (yes, no), hearing impairment (yes, no), stroke (yes, no), heart disease (yes, no), and hemoglobin (continuous).

**Table S4** Multinomial logistic regression analysis for the associations of CircS with global cognitive trajectories (Sensitivity Analysis with Additional Covariates).

|  | Persistently low (vs persistently high) | | Persistently moderate (vs persistently high) | |
| --- | --- | --- | --- | --- |
|  | OR (95% CI) * | *p* value | OR (95% CI) * | *p* value |
| Global cognitive scores |  |  |  |  |
| Crude model | 1.40[1.20-1.63] | <0.001 | 1.08[0.96-1.21] | 0.189 |
| Adjusted model* | 1.31[1.09-1.57] | 0.004 | 1.06[0.93-1.21] | 0.377 |

95%CI: 95% confidence interval;

*Adjusted for age at baseline (continuous), gender (male, female), education (primary school or below, junior high school to high school, undergraduate or above), marital status (married, unmarried), residence (urban, rural), smoking (yes, no), drinking (yes, no), body mass index (< 18.5, 18.5–23.9, 24.0–27.9, ≥ 28.0 kg/m2), restriction on activities of daily living (yes, no), visual impairment (yes, no), hearing impairment (yes, no), stroke (yes, no), heart disease (yes, no), hemoglobin (continuous), physical activity level (low, moderate, or high), social activity (none, moderate, or high), use of sleep medication (yes/no), use of antidepressant medication (yes/no), and habitual napping (0min, 1-60min, >60min).

**Table S5** causal forest analysis with additional covariates: average treatment effects (ATE) and distribution of individual treatment effects (ITE) across the three cognitive trajectories.

| Cognitive Trajectory | ATE (95%CI) | SD of ITE | Range of ITE |
| --- | --- | --- | --- |
| Low | 0.0312 (0.0111-0.0513) | 0.0142 | -0.0119 to 0.0861 |
| Moderate | -0.0035 (-0.0306 to 0.0237) | 0.0230 | -0.0955 to 0.0586 |
| High | -0.0311 (-0.0558 to -0.0064) | 0.0252 | -0.1028 to 0.0802 |

**Table S6** Multinomial logistic regression analysis for the associations of CircS with the global cognitive trajectory (Multiple Imputation on missing CircS)

|  | Persistently low (vs persistently high) | | Persistently moderate (vs persistently high) | |
| --- | --- | --- | --- | --- |
|  | OR (95% CI) * | *p* value | OR (95% CI) * | *p* value |
| Global cognitive scores |  |  |  |  |
| Crude model | 1.17[1.06-1.29] | 0.002 | 0.98[0.92-1.05] | 0.587 |
| Adjusted model* | 1.13[1.01-1.26] | 0.027 | 1.01[0.94-1.09] | 0.729 |

95%CI: 95% confidence interval;

*Adjusted for age at baseline (continuous), gender (male, female), education (primary school or below, junior high school to high school, undergraduate or above), marital status (married, unmarried), residence (urban, rural), smoking (yes, no), drinking (yes, no), body mass index (< 18.5, 18.5–23.9, 24.0–27.9, ≥ 28.0 kg/m2), restriction on activities of daily living (yes, no), visual impairment (yes, no), hearing impairment (yes, no), stroke (yes, no), heart disease (yes, no), and hemoglobin (continuous).

**Table S7** causal forest analysis with multiple imputation on missing CircS: average treatment effects (ATE) and distribution of individual treatment effects (ITE) across the three cognitive trajectories.

| Cognitive Trajectory | ATE (95%CI) | SD of ITE | Range of ITE |
| --- | --- | --- | --- |
| Low | 0.0209 (0.0096-0.0322) | 0.0236 | -0.0744 to 0.1348 |
| Moderate | -0.0022 (-0.0189 to 0.0146) | 0.0351 | -0.1329 to 0.1325 |
| High | -0.0199 (-0.0364 to -0.0035) | 0.0342 | -0.1672 to 0.1143 |

**Table S8** Logistic regression analysis for the associations of CircS with cognitive transition based on individual slopes.

|  | Cognitive Decline (vs Cognitive Stability or Improvement) | |
| --- | --- | --- |
|  | OR (95% CI) * | *p* value |
| Global cognitive scores |  |  |
| Crude model | 1.05[0.94-1.16] | 0.411 |
| Adjusted model* | 1.04[0.92-1.17] | 0.528 |

95%CI: 95% confidence interval;

*Adjusted for age at baseline (continuous), gender (male, female), education (primary school or below, junior high school to high school, undergraduate or above), marital status (married, unmarried), residence (urban, rural), smoking (yes, no), drinking (yes, no), body mass index (< 18.5, 18.5–23.9, 24.0–27.9, ≥ 28.0 kg/m2), restriction on activities of daily living (yes, no), visual impairment (yes, no), hearing impairment (yes, no), stroke (yes, no), heart disease (yes, no), and hemoglobin (continuous).

**Supplementary Method 1: Detailed Methodology for Cognitive Trajectory Modeling**

**S1. Model Overview: Latent Class Growth Modeling (LCGM)**

In this study, we employed **Latent Class Growth Modeling (LCGM)**, a person-centered longitudinal data analysis technique, to identify unobserved (latent) subpopulations within our study sample that share similar developmental trajectories of cognitive function over time. Unlike growth curve models that estimate a single average trajectory for the entire population, LCGM acknowledges population heterogeneity by grouping individuals into distinct classes, each defined by a unique trajectory shape (e.g., intercept and slope).

The core idea of LCGM is that the overall population is a mixture of several underlying homogeneous groups. The model estimates the probability of belonging to each trajectory class and the shape of the trajectory for each class simultaneously.

**S2. Model Specification**

**S2.1. Outcome Variable and Timescale**

- The **outcome variable** was the global cognitive score, measured repeatedly across up to four waves of the CHARLS study (2011, 2013, 2015, 2018).
- **Age in years** was used as the **timescale** for modeling the trajectories, as it is a fundamental biological driver of cognitive aging. This approach aligns the trajectories with the physiological process of aging rather than time since the study began.

**S2.2. Functional Form of Trajectories**
We allowed for both linear and quadratic (non-linear) trends of cognitive scores over age within each class. The form of the trajectory for an individual in class $k$ can be expressed as:

$$Y_{it\mid k}=\beta_{0k}+\beta_{1k}(Age_{it})+\beta_{2k}(Age_{it}^{2})+\epsilon_{it}$$

where:

- $Y_{it\mid k}$ is the cognitive score for individual $i$ at time $t$, given membership in class $k$.
- $\beta_{0k}$ is the intercept (initial status) for class $k$.
- $\beta_{1k}$ and $\beta_{2k}$ are the linear and quadratic slope parameters, respectively, for class $k$.
- $Age_{it}$ is the age of individual $i$ at time $t$.
- $\epsilon_{it}$ is the time-specific residual, assumed to be normally distributed.

**S2.3. Model Estimation**
Models were estimated using full-information maximum likelihood (FIML) to handle missing data under the missing-at-random (MAR) assumption. This method utilizes all available data from each participant, including those with incomplete follow-up.

**S3. Model Selection and Fit Criteria**

To determine the optimal number of cognitive trajectory classes, we estimated a series of models, starting with 1-class (no heterogeneity) and incrementally increasing to 5 classes. The optimal model was selected based on a combination of statistical fit indices, interpretability, and clinical plausibility, guided by established recommendations in the literature.

The following fit indices were used for formal comparison:

1. **Bayesian Information Criterion (BIC) and Sample-Adjusted BIC (SABIC)**: Lower values indicate a better balance between model fit and parsimony. SABIC is often preferred for mixture models as it is less sensitive to sample size.
2. **Akaike's Information Criterion (AIC)**: Lower values indicate better fit, though it tends to favor more complex models.
3. **Entropy**: A measure of classification uncertainty, ranging from 0 to 1. Values closer to 1 indicate clear, well-separated classes. An entropy value > 0.7 is generally considered acceptable.
4. **Class Size**: The proportion of the sample in the smallest class should be sufficient for meaningful inference, typically no less than 5% of the total sample.

The model selection process was not automated but involved a careful evaluation of all the above criteria. For instance, a model with a lower BIC but an AvePP below 0.7 for any class or a class containing less than 5% of participants was considered suboptimal.

**S4. Model Fitting Procedure**

1. We fitted separate models for 1, 2, 3, 4, and 5 latent classes.
2. For each class solution (e.g., 3-class), we compared models with linear only versus linear + quadratic trends.
3. The fit statistics for all competing models were tabulated (as shown in Table 2 of the main text).
4. Based on the joint evaluation of SABIC, entropy, and AvePP, the **3-class model with quadratic trends** was selected as the optimal representation of the data. Although the 4-class model had a slightly lower BIC, its average posterior probability fell below the 0.7 threshold, indicating poor class separation.
5. Finally, each participant was assigned to the trajectory class for which they had the highest posterior probability of membership.

**S5. Software Implementation**

All LCGM analyses were performed using the lcmm package (Version 2.0.2) in **R version 4.4.2**. Multiple random starting values were used for each model to ensure that the solution represented a global, rather than local, maximum of the likelihood function.

**Supplementary Method 2: Detailed Description of the Causal Forest Methodology**

**S1. Theoretical Framework: From Average to Heterogeneous Treatment Effects**

In traditional observational studies, the focus is often on estimating the **Average Treatment Effect (ATE)**, which represents the average causal effect of an exposure (e.g., Circadian Rhythm Syndrome, CircS) across the entire study population. However, the ATE may mask substantial variation in how different individuals or sub-groups respond to the exposure. This variation is known as **Heterogeneous Treatment Effects (HTEs)**.

Our goal is to estimate the **Conditional Average Treatment Effect (CATE)**, which is the causal effect conditioned on a set of covariates $X$. Formally, for an individual $i$ with characteristics $X_{i}=x$, the CATE is defined as:

$$\tau(x)=E[Y_{i}(1)-Y_{i}(0)\mid X_{i}=x]$$

where $Y_{i}(1)$ and $Y_{i}(0)$ are the potential outcomes under treatment (CircS present) and control (CircS absent) states, respectively.

The **causal forest** is a non-parametric machine learning method designed specifically for estimating HTEs and inferring CATEs from observational data.

**S2. Causal Forest Algorithm Specification**

The causal forest is an ensemble method built from a collection of **causal trees**. We implemented the algorithm using the grf package in R. The key features of our implementation are outlined below.

**S2.1. Base Learner: Causal Trees**

- Each causal tree partitions the covariate space into sub-populations (leaves) where the treatment effect is assumed to be approximately constant.
- The splitting rule at each node of the tree is designed to maximize the heterogeneity in the treatment effect between the two resulting daughter nodes, rather than predicting the outcome $Y$ itself.

**S2.2. Ensemble: From Trees to Forest**

- We grew an ensemble of $B=10,000$ causal trees.
- Each tree was trained on a random subsample of the data (drawn without replacement) to ensure tree diversity and de-correlate their predictions.

**S2.3. Honesty**

- We employed **honest estimation**, a critical feature for obtaining statistically valid confidence intervals.
- For each tree, the data used for growth (splitting the nodes) and the data used for estimation (calculating the treatment effect within leaves) were disjoint. Specifically, we used a 50%/50% split.

**S2.4. Parameter Tuning**
To prevent overfitting and ensure robust splits, we applied the following regularization parameters:

- **Imbalance penalty (**$\lambda$**)**: Set to 0.1. This penalizes splits that lead to daughter nodes with very different sizes or different proportions of treated and control units, favoring more balanced splits.
- **Significance level (**$\alpha$**)**: Set to 0.05. A covariate must have a statistically significant association with the treatment effect (p < 0.05) to be considered for a split, reducing noise.

**S3. Data Preparation and Model Fitting**

**S3.1. Study Population and Variables**

- The analysis included 6,218 participants from the CHARLS cohort with complete data on CircS, cognitive trajectories, and all covariates.
- **Treatment Variable (**$W$**)**: CircS, defined as a binary variable (1 = present, 0 = absent).
- **Outcome Variable (**$Y$**)**: Cognitive trajectory membership. As the outcome was multi-class ("persistently high", "persistently moderate", "persistently low"), we ran three separate binary causal forest models. For each model, the outcome was dichotomized (e.g., for the "persistently low" model, 1="persistently low", 0="all other trajectories").
- **Covariates (**$X$**)**: We included 14 pre-specified covariates: age, gender, residence, education level, marital status, smoking status, drinking status, history of coronary heart disease, history of stroke, hemoglobin level, body mass index (BMI), visual impairment, hearing impairment, and activities of daily living (ADL) score.

**S3.2. Estimation Procedure**
For each individual $i$, the causal forest provides an estimate of the **Individual Treatment Effect (ITE)**, $\hat{\tau}(x_{i})$. The model does this by:

1. Finding other individuals who are similar to individual $i$ in terms of their covariates $X$ (their "neighbors" in the covariate space).
2. Calculating the treatment effect within this local neighborhood.
3. Aggregating these local estimates across all trees in the forest to produce a robust ITE for individual $i$.

The **Average Treatment Effect (ATE)** is then computed as the mean of all individual ITEs:

$$\hat{\tau}_{ATE}=\frac{1}{n}\sum_{i=1}^{n} \hat{\tau}(x_{i})$$

**S4. Model Evaluation and Validation**

**S4.1. Calibration Assessment**
We assessed the calibration of the causal forest using the **best linear predictor (BLP) test**.

- This test regresses the observed outcome on the forest's predicted treatment effects.
- A well-calibrated model should have an intercept close to 0 and a slope for the predicted treatment effects close to 1.
- A statistically significant slope ($p<0.05$) indicates that the model has successfully captured genuine treatment effect heterogeneity.

**S4.2. Quantifying Heterogeneity**

- The degree of HTE was quantified as the **standard deviation of the estimated ITEs** across the population.
- We also visually inspected the distribution of ITEs using density plots.

**S4.3. Variable Importance**
The contribution of each covariate to explaining the heterogeneity was assessed based on:

- **Frequency**: How often a variable was used in splitting across all trees in the forest.
- **Impurity Decrease**: The total reduction in the variance of the treatment effect estimates attributable to splits on that variable.

**S4.4. Sensitivity and Robustness Analyses**

- **Model Stability**: We re-estimated all models using 5,000 trees to ensure that the ATE and variable importance rankings were consistent and not dependent on the specific number of trees.
- **Effect Stability**: We compared the ATE estimates from the causal forest with those from traditional regression models as a sanity check.

**S5. Software and Reproducibility**

All causal forest analyses were performed using **R version 4.4.2**. The primary package used was grf (Version 2.3.0). Supplementary packages for data manipulation and visualization included ggplot2, patchwork, and dplyr. To ensure full reproducibility, the random seed was set to 123 prior to all analyses.
